# Highest risk abandoned, lost and discarded fishing gear

**DOI:** 10.1038/s41598-021-86123-3

**Published:** 2021-03-30

**Authors:** Eric Gilman, Michael Musyl, Petri Suuronen, Milani Chaloupka, Saeid Gorgin, Jono Wilson, Brandon Kuczenski

**Affiliations:** 1 The Nature Conservancy, California Oceans Program, Santa Barbara, USA; 2 Pelagic Research Group, Honolulu, USA; 3 Natural Resources Institute Finland (Luke), Helsinki, Finland; 4 Ecological Modelling Services Pty Ltd & Marine Spatial Ecology Lab, University of Queensland, Brisbane, Australia; 5 Department of Fisheries, College of Fisheries and Environment, Gorgan University of Agricultural Sciences and Natural Resources, Gorgān, Iran; 6 Bren School of Environmental Science & Management, University of California Santa Barbara, Santa Barbara, USA

**Keywords:** Ecology, Biodiversity

## Abstract

Derelict abandoned, lost and discarded fishing gear have profound adverse effects. We assessed gear-specific relative risks from derelict gear to rank-order fishing methods based on: derelict gear production rates, gear quantity indicators of catch weight and fishing grounds area, and adverse consequences from derelict gear. The latter accounted for ghost fishing, transfer of microplastics and toxins into food webs, spread of invasive alien species and harmful microalgae, habitat degradation, obstruction of navigation and in-use fishing gear, and coastal socioeconomic impacts. Globally, mitigating highest risk derelict gear from gillnet, tuna purse seine with fish aggregating devices, and bottom trawl fisheries achieves maximum conservation gains. Locally, adopting controls following a sequential mitigation hierarchy and implementing effective monitoring, surveillance and enforcement systems are needed to curb derelict gear from these most problematic fisheries. Primary and synthesis research are priorities to improve future risk assessments, produce the first robust estimate of global derelict gear quantity, and assess the performance of initiatives to manage derelict gear. Findings from this first quantitative estimate of gear-specific relative risks from derelict gear guide the allocation of resources to achieve the largest improvements from mitigating adverse effects of derelict gear from the world’s 4.6 million fishing vessels.

## Introduction

Over the past decade there has been increasing international recognition of the need for multilateral efforts to address transboundary adverse ecological and socioeconomic effects of abandoned, lost and discarded fishing gear (ALDFG), also called derelict fishing gear^1, 2^. The amount, distribution and effects of ALDFG have likely risen in recent decades with the rapid expansion of fishing effort and fishing grounds and the transition to synthetic, less-expensive, more durable and more buoyant materials used for fishing gear^3–6^.

Most marine debris is now made of synthetic plastics, some readily visible, some microscopic^7–9^. About 10 million tonnes of plastic may enter oceans annually^10–13^ and this amount will likely substantially increase over the next decade^14^. Most marine debris originate from land-based sources, but at local-scales, sources are highly variable^4, 10^. Currents and wind can disperse floating debris over vast distances and can cause floating debris to concentrate into vast garbage patches in oceanic gyres^15^. Debris may oscillate in the water column, sinking once biofouling increase its density, and rising with a decrease in foulants^16, 17^. Some floating debris washes ashore while most marine plastic sinks to the deep seabed^3, 7–9, 18^. As with floating debris, bottom thermohaline currents can concentrate microplastics in benthic habitats at sites that may coincide with biodiversity hotspots^19^. Plastics can persist with low degradation in marine environments below the photic zone, especially on the deep seabed^8, 9, 16^.

There is extremely limited understanding of the life cycle and end-of-life management of non-biodegradable fishing gear. Since the invention of fully synthetic plastic over a century ago and the advent of mass production of plastic products in the 1950s, plastics are now essential for countless applications^12^. However, the use of plastic for short-term applications in linear instead of circular economies, including by most marine fisheries, contributes to myriad ecological and socioeconomic problems^3, 20^.

Derelict fishing gear from the world’s estimated 4.6 million marine fishing vessels^21^ is a profound stressor of coastal and marine ecosystems^21–24^. Given that fishing gear is designed to catch marine organisms, relative to other sources of marine debris, ALDFG from some gear types, under certain conditions, can have an extremely long duration of ghost fishing efficiency, resulting in substantial fishing mortalities (Table 1). This includes a high catch risk for both market species and bycatch, including vulnerable species, which is the focus of political and media attention. Other adverse consequences include transporting and transferring toxins and microplastics into marine food webs; transporting invasive alien species; distributing microalgae that may cause harmful algal blooms; altering and damaging habitat; obstructing in-use fishing gear and navigation; creating safety risks at sea; and reducing the socioeconomic value of coastal and nearshore habitats (Table 1). ALDFG can also have positive ecological consequences, such as providing artificial habitat and a meeting point to re-form fish schools; and socioeconomic benefits such as repurposing for various applications^25–27^.

**Table 1:** Adverse consequences of ALDFG, factors that determine whether the adverse effect occurs and the severity of the effect, and metrics for gear-specific assessment.

Consequence	Factors affecting the occurrence and magnitude of the adverse consequence	Metrics for gear-specific ALDFG assessment	Citations
Ghost fishing, ingestion of ALDFG components	•Initial ghost fishing efficiency. Determined in part by whether: (i) the gear was abandoned or lost after being set, or discarded; and (ii) does the catching process cease or continue once the gear is lost or abandoned. In general, active gears, such as purse seines and trawls, typically cease to have catching efficiency once detached from the vessel. •Duration of ghost fishing efficiency, including risk of ingestion of components of ALDFG, which is determined by: (i) whether self-baiting occurs (organisms captured in the derelict gear attract predators and scavengers which subsequently are caught), (ii) whether gear incorporates designs intended to reduce ghost fishing efficiency—such as degradable escape panels and cords in traps and biodegradable drifting fish aggregating devices, and (iii) the conditions of the location where the ALDFG occurs. Local conditions that influence ghost fishing efficiency and duration include: (i) is the substrate protected and have 3-dimensional features vs. open and flat; (ii) exposure to environmental forces that can disable the derelict gear; (iii) exposure to vessels and mobile fishing gears that can disable the derelict gear; (iv) local abundance of biofouling organisms, debris and particulate matter; and (v) local abundance of organisms susceptible to capture in the gear. •Vulnerability and socioeconomic value of species subject to ghost fishing removals and ingestion of ALDFG, determined by the fishing efficiency and duration of fishing efficiency of the derelict gear; and local abundance of species susceptible to capture in the derelict gear.	•Risk of ghost fishing mortality when gear initially becomes derelict •Duration of ghost fishing efficiency, accounting for effects of self-baiting, prevalence of use of designs intended to reduce ghost fishing efficiency, environmental conditions that could disable the gear, exposure to vessels and in-use mobile gear, local abundance of species susceptible to capture • Vulnerability and socioeconomic value of species susceptible to ghost fishing, including ingestion of components of ALDFG	^4, 23, 29, 72–77^
Dispersal and transfer of toxins and microplastic into marine food webs	•The proportion of the volume of derelict gear that is made of plastic, and mass of plastic per unit of derelict gear (e.g., per pot, per panel of gillnet), partly explains the magnitude of microplastic and toxin inputs to marine food webs. We do not use proportion of the mass of derelict gear as a metric, as this would result in relatively low risk scores for gears with relatively heavy non-plastic components (e.g., gillnet anchors and leadlines, longline branchline weights, hooks, snaps and swivels). Plastics leach chemical pollutants. Chemical sorption of persistent organic pollutants (POPs), including polychlorinated biphenyls (PCB), pesticides, and polycyclic aromatic hydrocarbons (PAHs) to plastic marine debris increase with time in seawater, and when ingested, the chemicals transfer from the plastic to the marine organism. •Whether macro-plastic components of derelict gear break down into micro- and nanoplastic-sized pieces depends on the degradation rate of the type of plastic used in the derelict gear, and the environment where the debris is located, such as at the sea surface where it is exposed to ultraviolet (UV) radiation, heat, mechanical stress from wave and wind energy, and relatively high microorganism and macrofauna local abundance, vs. buried in anoxic mud on the deep seafloor. •Whether components of derelict gear transport and release toxins that are incorporated into food webs, and which toxins are transported and released, depends on the location and movement of the derelict gear, and the types of plastic materials and toxic metals (e.g., lead, zinc, cadmium) that are used for gear components. We do not include indicators for the types of metal or types of plastics because materials used can be highly variable. There is also incomplete understanding of the chemicals associated with different types of plastic and the species-specific risks of toxic effects from individual and combinations of chemicals •Whether microplastics and leached toxins enter marine food webs depends on the fate of the debris—does it end up buried on the seafloor in the deep ocean, on a hard bottom, shallow, coastal habitat, floating at the sea surface, etc.	•Proportion of the volume of derelict gear that is made of plastic •Exposure to forces (abrasion, reactions from exposure to UV radiation—photolysis, photo-oxidation, thermo-oxidation, biodegradation) that cause plastic gear components to break down into microplastic Relative productivity of the habitats(s) where the ALDFG occurs (an indicator of the relative risk of incorporation of toxins and microplastic into food webs)	^8, 18, 22, 76, 78–83^
Dispersal of invasive alien species (IAS) and microalgae that cause harmful algal blooms (HABs)	•Whether the derelict gear sinks or floats partly explains the risk of spreading IAS and species of microalgae that cause HABs. •The region(s) where the gear is used partly explains risk. However, given the broad geographical distribution of documented HABs, we assume that all areas have equal risk of exposure to the dozen or so species of dinoflagellates responsible for regional spreading of HABs.	•Does derelict gear initially float or sink	^25, 84–86^
Habitat alteration and degradation	•The habitat types where the derelict gear occurs •The risk that the derelict gear will damage habitat, such as through scouring, abrading, smothering or altering the habitat’s structure	•Risk of damaging sensitive habitats	^26, 41, 74^
Obstruction of in-use fishing gear and navigation, creation of safety risks at sea	•The risk that derelict gear will encounter marine vessels and in-use fishing gear, and cause fouling, is explained in part by: Whether the derelict gear floats or sinks, as floating debris has a higher risk of obstructing marine vessels •Whether the derelict gear occurs in areas with marine vessel traffic •Whether there is aerial and vertical overlap between the derelict gear and in-use fishing gear •Whether the derelict gear materials risk fouling vessels and in-use fishing gear •The relative visibility of surface and subsurface derelict gear	•Does derelict gear initially float •Derelict gear spatial and vertical overlap with in-use fishing gear •Derelict gear materials’ risk of fouling vessels and in-use fishing gear •Derelict gear visibility	^4, 24, 64, 87^
Reduced socioeconomic, aesthetic and use values of coastal and nearshore areas	•Risk that the derelict gear will ground on coastlines and nearshore habitats used for human activities such as recreation, tourism, education and research, and residential and commercial purposes, which is explained in part by whether the fishing grounds are located in nearshore areas, and whether the derelict gear initially floats and thus has the potential to be transported to nearshore and coastal habitats. •The proportion of the derelict gear that might occur on coastlines and nearshore areas that is not made of natural and biodegradable materials. Debris made of natural and biodegradable materials may be less disruptive aesthetically and persist for a shorter duration than synthetic debris. Biodegradable ALDFG may also have a shorter duration of ghost fishing efficiency, reducing adverse effects on aesthetic and use values, for example, of popular dive sites.	•Risk derelict gear will occur on coastlines and nearshore habitats with socioeconomic value •Proportion of the volume of derelict gear that might occur on coastal and nearshore areas not made of natural and biodegradable materials	^41, 88, 89^

The United Nations Sustainable Development Goal 14.1 aims to, “by 2025, prevent and significantly reduce marine pollution of all kinds, particularly from land-based activities, including marine debris and nutrient pollution”^28^. There is extremely limited information available on the magnitude of marine debris, including ALDFG^1, 29^. This prevents measuring progress towards meeting the UN goal. A 2009 report by the Food and Agriculture Organization of the United Nations and United Nations Environment Programme conjectured that ALDFG is < 10% of the volume of total marine litter, and cited a 1975 estimate (Tables 8–13 in^30^) that 6.36 million t of marine litter is leaked annually^4^, producing the rough estimate that 640 thousand t of ALDFG is produced annually. This 5-decades-old rough estimate has been repeatedly referenced (e.g.,^31–33^) despite explicit caveats of the uncertainty of the approximations stated in the source publications^4, 30^.

More recent estimates of the quantity of global ALDFG are likewise highly uncertain (e.g., 1.14 Mt of derelict gear leaked annually^11^). Studies have estimated loss rates, with large uncertainty, for small proportions of fishing gears and regions^4, 29, 34^. Furthermore, there is extremely limited understanding of gear-specific relative risks from ALDFG. Substantially more certain and contemporary estimates of gear-specific rates, magnitude and adverse effects of ALDFG are needed to serve as a benchmark against which to measure the performance of management interventions^1^.

This study contributes to filling these priority knowledge gaps. We ranked fishing gears used in commercial marine capture fisheries according to their global adverse effects from ALDFG. The gear-specific relative risks were estimated using derelict gear leakage rates, catch levels, geo-spatial area of fishing grounds and adverse ecological and socioeconomic impacts from a unit of derelict gear. Findings guide the allocation of resources for management interventions to prevent and reduce this particularly problematic component of global marine litter.

## Results

**Figure 1:**
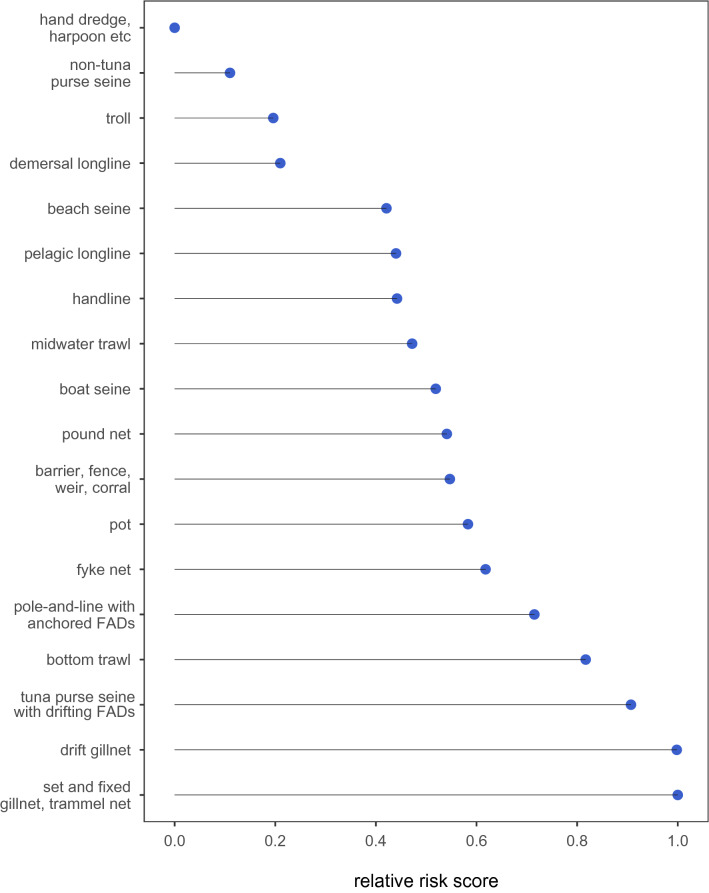
Gear-specific relative risk from abandoned, lost and discarded fishing gear (ALDFG). From the top of the *y*-axis, fishing gears are listed from lowest overall relative risk score, which accounts for: (**a**) rate of production of ALDFG, (**b**) fishing effort (accounts for gear-specific weight of total catch and geospatial area of fishing grounds), and (**c**) adverse ecological and socioeconomic impacts of ALDFG (accounts for: ghost fishing, dispersal and transfer of toxins and microplastic into marine food webs, dispersal of invasive alien species and microalgae that cause harmful algal blooms, habitat degradation, obstruction and safety risks to navigation and in-use fishing gear, and reduced socioeconomic, aesthetic and use values of coastal and nearshore habitats). The higher the relative risk score, the larger the amount of global adverse effects from ALDFG the gear is estimated to be causing, based on the quantity of derelict gear that gear leaks into the oceans and the relative adverse effects caused by ALDFG from that gear type. The first gear category includes boat and shore-based hand dredge, harpoon, spear, lance, tongs, rakes, and hand-collected, including diving.

Figure 1 presents gear-specific scores of overall relative risks from ALDFG. The five highest-risk gears with scores in the 75% quantile and above (RR > 0.70) were: set and fixed gillnet and trammel net, drift gillnet, tuna purse seine with FADs, bottom trawl and pole-and-line with anchored FADs. The five lowest-risk gears with scores in the 25% quantile and below (RR < 0.43) were: beach seine, demersal longline, troll, non-tuna purse seine, and miscellaneous (hand dredge, harpoon etc.).

Supplementary Material Section S3 contains gear-specific estimates of adverse ecological and socioeconomic effects from ALDFG. Table 2 contains gear specific values for each of the terms that were used to calculate gear-specific overall relative risk scores presented in Fig. 1. Table 2 summarizes gear-specific scores for: (a) rate of production of ALDFG, (b) catch, and (c) geo-spatial area of fishing grounds, which combined indicates the gear-specific relative magnitude of ALDFG. Table 2 also summarizes gear-specific scores for relative adverse ecological and socioeconomic effects from ALDFG.

**Table 2:** Gear-specific relative risk scores, on a scale of 0 to 1, with 0 being no risk and 1 highest risk, for rates of the production of ALDFG (*R*), global annual catch (*C*), area of fishing grounds (*A*), and adverse ecological and socioeconomic outcomes from ALDFG (*O*).

Fishing gear	*R*	*C*	*A*	*O*
Barrier, fence, weir, corral	0.12	0.02	0.05	0.55
Fyke net	0.19	0.02	0.05	0.62
Gillnet, drift	0.15	0.12	0.06	1.00
Gillnet, set and fixed; trammel net; combination gillnet/trammel net	0.39	0.24	0.32	0.95
Hand dredge, harpoon, spear, lance, tongs, rakes, hand-collected, including diving—shore- and boat-based	0.001	0.05	0.06	0.003
Handline, pelagic and bottom	1.00	0.16	0.87	0.19
Longline, demersal	0.88	0.02	0.01	0.21
Longline, pelagic	0.11	0.06	0.45	0.43
Pole-and-line, including anchored FADs	0.96	0.01	0.87	0.51
Pot	0.89	0.04	0.05	0.57
Pound net	0.12	0.02	0.05	0.54
Purse seine, non-tuna	0.07	0.49	0.21	0.10
Purse seine, tuna, including drifting and anchored FADs	0.75	0.05	1.00	0.72
Seine, beach	0.21	0.01	0.02	0.42
Seine, boat	0.24	0.10	0.07	0.51
Trawl, bottom	0.16	1.00	0.21	0.78
Trawl, midwater	0.49	0.39	0.09	0.42
Troll	0.16	0.01	0.87	0.16

The gears with the two highest scores for overall relative risk from ALDFG were set and fixed gillnets and trammel nets and drift gillnets (Fig. 1). These gears had median scores of assessed gears for ALDFG rates and effort/quantity of gear, and had the two highest scores for relative adverse outcomes (Table 2). The gears with the two lowest overall relative risk scores were hand dredges, harpoon and other gears, and non-tuna purse seines (Fig. 1). These gears had the two lowest scores for both relative ALDFG rate and adverse effects, and median scores for effort/quantity of gear (Table 2).

## Discussion

### Most problematic fishing methods based on ALDFG relative risks

This study presents the first quantitative assessment of gear-specific relative risks from ALDFG. Findings accounted for the: (a) derelict gear leakage rate; (b) fishing gear quantity indicators of catch and area of fishing grounds; and (c) adverse consequences from ALDFG. Maximum global conservation gains can be achieved through focusing ALDFG mitigation efforts on the fishing gears with the highest overall relative risk. Set and fixed gillnets and trammel nets, drift gillnets, gears using drifting and anchored FADs (tuna purse seines and pole-and-lines), and bottom trawls were the five most problematic gears on a global scale. This was followed by traps (fyke nets, pots, barriers, fences, weirs, corrals and pound nets).

The overall *RR* score indicates a fishing gear’s relative degree of total adverse effects from ALDFG, accounting for the quantity of ALDFG produced by that gear (estimated from the ALDFG leakage rate and indices of fishing gear quantity of catch and area of fishing grounds), and the adverse consequences that result from ALDFG from that gear type relative to other gears. Globally, gillnets have the highest risks from ALDFG, while hand dredges and harpoons were least problematic.

The focus of local management interventions to address problematic derelict fishing gear will be dictated by the specific context. Locally, adopting ALDFG controls following a sequential mitigation hierarchy and implementing effective monitoring, surveillance and enforcement systems are needed to curb derelict gear from these most problematic fisheries. This includes accounting for which fishing gears are predominant and the existing fisheries management framework. For example, a site may have pot and tuna purse seine anchored FAD fisheries. The purse seine fishery has a higher relative risk globally. However, a fisheries management system may have effective ALDFG preventive methods in place for this fishery, such as a high rate of detection and recovery of anchored FADs when they break from moorings, and minimization methods, such as prescribing the use of only non-entangling and biodegradable FAD designs to minimize adverse effects from derelict FADs^35, 36^. But there may be minimal measures in place to monitor and manage ALDFG from pots. In this hypothetical example, it would be a higher priority locally to improve ALDFG management for the pot fishery.

### Priority data quality improvements

There are several priorities for data quality improvement to increase the certainty of future assessments. Given substantial deficits both in estimates of gear-specific quantity/effort and ALDFG rates, it is not yet possible to produce a robust contemporary estimate to replace the ca. five decades-old crude estimate of the magnitude of the annual quantity of leaked ALDFG^4, 30^. More robust estimates of ALDFG rates are needed for all gear types. Gear-specific estimates have low certainty due to small numbers of studies and sample sizes. Many compiled records estimate only one ALDFG component, typically only loss rates, and therefore may substantially underestimate total ALDFG rates. Most records are dated and may not accurately characterize contemporary rates. There is geographical sampling bias with estimates being primarily derived from the northern hemisphere. Furthermore, many estimates were derived from expert surveys (Supplementary Material Table S1), which have a higher risk of error and bias than approaches higher on the evidence hierarchy^37^. Substantially more primary studies with robust designs are needed.

An expanded meta-analysis on gear-specific ALDFG rates is an additional priority, once sufficient sample sizes of robust studies accumulate. The statistical modeling approach used by Richardson et al.^34^ could be readily improved by using (1) a random-effects instead of a fixed effects structure to account for study-specific heterogeneity, and (2) a more appropriate model likelihood, such as zero-inflated Beta likelihood, to account for the zero values in the dataset^38^. Due to larger sample sizes and the number of independent studies, meta-analyses can produce estimates with increased accuracy, with increased statistical power to detect real effects. By synthesizing estimates from an assortment of independent, small and context-specific studies, pooled estimates from random-effects meta-analyses are generalizable and therefore relevant over diverse settings^39^. The strength of conclusions of hypotheses based on a single study can vary. This is because a single study can be context-specific, where true results may be affected by conditions specific to that single study, such as the species involved and environmental conditions, that cause the results from the single study to not be applicable under different conditions. A single study may also fail to find a meaningful result due to small sample sizes and low power. However, robust synthesis research, including meta-analysis, is more precise and powerful once a sufficient number of similar studies have accumulated, and therefore investing in more primary ALDFG studies is a high priority.

For some gear types and fisheries, estimated ALDFG rates may overestimate adverse effects when gear that is abandoned, lost or even discarded does not become derelict because another fishing vessel continues to use the gear. For example, gear that is lost by theft remains in use. Macfadyen et al.^4^ explained that theft was likely a minor contribution to ALDFG, occurring, for instance, in inshore fishing grounds where static commercial fishing gear and recreational marine activities conflict. However, fishing gear theft may be prevalent in some developing country fisheries (e.g., Cambodian crab traps^40^). And, there is one gear type where theft has become a globally prevalent, routine and largely accepted practice: Tuna purse seine vessels routinely exchange satellite buoys attached to drifting FADs that they encounter at sea. The stolen FAD, lost by the previous vessel that had been tracking its position, remains in-use and not derelict, although it may eventually become derelict^41, 42^. Furthermore, because ALDFG leakage rates may be highest in illegal and unregulated fisheries^4^, if only legal fisheries are sampled, then this may produce underestimates. Thus, accounting for theft and illegal and unregulated fishing would increase the certainty of estimates of ALDFG leakage rates for some fishing gear types.

The 20% ALDFG global production rate value used for anchored FADs by pole-and-line fisheries was likely an underestimate. We relied on a single value from the contemporary Maldives pole-and-line fishery’s government-owned and -managed network of anchored FADs. This fishery underwent a substantial reduction in anchored FAD loss rate, from 82 to 20%, by improving designs and a government incentive program that pays fishers to retrieve FADs when they break from their moorings^35, 43^. For comparison, describing Indonesia’s pole-and-line fishery’s anchored FADs, Widodo et al.^44^ stated: “Inaccuracy of number and position of FADs in the fishing ground are the outstanding issue facing by fisheries manager…This was largely the result of the current lack of effective systems of FAD registration and monitoring, and also because of the desire of fishing companies and vessel skippers to keep FADs position information confidential. [sic]”.

Proctor et al.^45^, who estimated that between 5000 and 10,000 anchored FADs are used in Indonesian tuna fisheries, also reported a lack of accurate estimates of the numbers and locations of anchored FADs due to ineffective implementation of the government registration system and to high loss rates, including from storms, strong currents, vandalism, vessel collisions and wear and degradation of the FADs. Using the estimated rates of (1) Shainee and Leira^43^ that 82% of anchored FADs were lost per year prior to the Maldivian government’s incentives program, which might accurately characterize the Indonesian and other anchored FAD networks used by pole-and-line fisheries, and (2) the 20% loss rate value from Adam et al.^35^, the posterior mean = 0.506 (95% HDI: 0.15–0.84). Thus, 51% might have been a more appropriate estimate for a global ALDFG production rate for pole-and-line anchored FADs. The Maldivian and Indonesian pole-and-line fisheries, which combined supply over half of global pole-and-line catch, rely heavily on anchored FADs, as do several other smaller pole-and-line fisheries (e.g., Solomon Islands, segments of the Japanese pole-and-line fleet)^35, 45–48^.

Units for ALDFG rates are highly variable. Records using different rates cannot be pooled for synthesis research^29, 34^. For example, some records reported rates of the percent of number of panels (sheets) or fleets (strings) of gillnets that were lost, while others reported the percent of the length or area of gillnets that were lost^29^. Similarly, for longline gear, some studies reported the percent of the length of the mainline, while others reported the percent of the number of branchlines/snoods that were lost^34^. Employment of agreed harmonized units for ALDFG rates are needed.

Future assessments could use a ratio of ALDFG risk-to-seafood production to assess gear-specific relative risks locally and globally, similar to assessments of vulnerable fisheries bycatch by using bycatch-to-target catch ratios^49^. This would enable the assessment of risk from ALDFG to be balanced against meeting objectives of food security and nutritional health.

### Relationship between alternative indices with the quantity of fishing gear

We used gear-specific annual catch and area of fishing grounds as indicators of the relative global amount of each gear that is used annually as two terms in the model to assess gear-specific relative risks from ALDFG. However, the assumption of a linear relationship between these indices and gear quantity is questionable for similar reasons that have been raised with the relationship between various indices of effort (number of fishing hours, number of vessels, engine power, vessel length, gross tonnage, gear size, hold capacity, as well as kWh) and catch. For example, the ratio of catch from one set by an anchoveta purse seiner to the volume or weight of the gear is likely substantially different than for pots or driftnets. Not only is the relationship between catch and amount of gear variable by gear type and target species, there is also high variability within gear types—by fishery and within fisheries—due to the broad range of factors that significantly explain fishing efficiency per unit of nominal effort^50, 51^. Similarly, the relationship between catch weight and number of fishing operations varies substantially across gear types. For example, an industrial tropical tuna purse seine vessel might have a total catch of about 37 t per set on a drifting FAD^27^ while a tuna pole-and-line vessel catches about 1 t per fishing day^52^.

Similarly, the relationship between the area of fishing grounds and amount of gear may vary substantially between gear types. A small number of vessels using a relatively small magnitude of active, mobile gear may have a much larger area of fishing grounds than a large number of vessels and shore-based fishers using a large amount of passive and static gears. For example, about 686 large-scale tuna purse seine vessels fish across the tropics^53^, while gillnets, which may be the most globally prevalent gear type, are used predominantly within 20 nm (37 km) of shore, most intensively in southeast Asia and the northwest Pacific^54^.

Fishing effort has also been estimated using engine power as well as by using energy expended, such as in kilowatt-hours (kWh), the product of the fishing time and engine power of a fishing vessel, including non-motorized vessels^55–57^. We did not use these metrics for effort because the correlation between rate of production of ALDFG and vessel engine power or kWh, including of non-motorized vessels (1.70 million of the estimated global 4.56 million fishing vessels^21^), has not been explored. In general, vessel power and power per unit of fishing period largely distinguishes between mobile and passive gears, where the former (e.g., trawls, dredges), use substantially more vessel power per weight of catch than passive gears (traps, gillnets). Also, estimates using these fishing effort metrics used a small number of aggregated gear categories and extrapolated estimates primarily from sampled developed world fisheries (however, see^56^). These effort indices would also prevent inclusion of shore-based fishing methods.

There have been recent gear specific estimates of effort, in units of time spent fishing and the estimated energy expended (fishing power * fishing time), using Automated Identification Systems (AIS) data, which are available for industrial fishing vessels, primarily using longlines, trawls and pelagic purse seines^6, 58^. AIS data provide coverage of the majority of large fishing vessels ≥ 24 m in overall length^58^. However, this accounts for only about 2% of the number of global fishing vessels (of an estimated 4.56 million global fishing vessels, about 67,800 are ≥ 24 m in length^21^).

### ALDFG monitoring, management and performance assessments

A sequential mitigation hierarchy of avoidance, minimization, remediation and offsets can be applied to manage ALDFG^29, 59^. Referring to the three components of relative risk assessed by this study, avoidance and minimization of risks from ALDFG is achieved by reducing the ALDFG leakage rate, fishing effort, and/or adverse consequences from derelict gear. Remedial methods reduce adverse effects, such as reducing ghost fishing by reducing the duration that ALDFG remains in the marine environment^1, 29, 60^. In general, preventative methods are more cost effective than remedial methods—it is less expensive to prevent gear abandonment, loss and discarding than it is, for example, to detect and then disable or remove derelict gear^61^. Methods to prevent ALDFG include, for instance, spatially and temporally separating passive and mobile fishing gears, having bottom trawlers avoid features that could snag the net such as by using high-resolution seabed maps, tracking the real-time position of unattended fishing gears using various electronic technologies, and using gear marking to identify the owner and increase the visibility of passive gears. Furthermore, because some remedial methods, such as using less durable materials for fishing gear components, can reduce economic viability and practicality, preventative methods and remediation through quick recovery of ALDFG may be more effective as well as elicit broader stakeholder support^29, 62^.

To assess the performance of global ALDFG management interventions against this study’s quantitative benchmark, substantial deficits in monitoring and surveillance of fisheries’ waste management practices must first be addressed^1^. Of 68 fisheries that catch marine resources managed by regional fisheries management organizations, 47 lack any observer coverage, half do not collect monitoring data on ALDFG, and surveillance and enforcement systems are rudimentary or nonexistent in many fisheries^1, 63^.

Findings from this quantitative, global assessment of ALDFG risks guide the allocation of resources to achieve the largest improvements from preventing and remediating derelict gear from the world’s 4.6 million fishing vessels. With improved data quality and governance frameworks for fishing vessel waste management, including ALDFG, we can expect reductions in ecological and socioeconomic risks from derelict gear.

## Methods

We used gear categories of the highest resolution possible based on the categories for which estimates were available for ALDFG leakage rates, catch levels and geo-spatial area of fishing grounds. This resulted in 18 fishing gear categories being included in the study:

- Gillnets and entangling nets: (1) drift gillnet; (2) set and fixed gillnet, trammel net, combination gillnet/trammel net
- Hook-and-lines: (3) bottom, midwater and surface handline; (4) demersal longline; (5) pelagic longline; (6) pole-and-line including fish aggregating devices (FADs); (7) troll.
- Miscellaneous: (8) hand dredge, harpoon, spear, lance, tongs, rakes, hand-collected (including diving)—shore- and boat-based
- Seine nets: (9) beach seine; (10) boat seine (Danish seine [anchor seining], Scottish seine [fly-dragging])
- Surrounding nets: (11) non-tuna purse seine; (12) tuna purse seine, including FADs
- Traps: (13) barrier, fence, weir, corral; (14) fyke net; (15) pot; (16) pound net
- Trawls: (17) midwater trawl; (18) bottom trawl.

About half of the global tropical tuna catch by purse seine fisheries is derived from sets on drifting FADs. Networks of anchored FADs (called rumpons in Indonesia, payaos in the Philippines), are also used by some tuna purse seine fisheries, primarily in nearshore waters in the western Pacific Ocean^27, 64, 65^. A majority of the catch from pole-and-line fisheries comes from fisheries where some of the fishing effort occurs on anchored FADs^35, 44–48, 66,89^. Some tuna pole-and-line fisheries also fish on drifting FADs, likely deployed by tuna purse seine fisheries^44, 47, 66, 67^. While FADs are also used by some handline, jig, troll and driftnet fisheries^44–46, 64, 68^, this study did not include FADs as part of the assessment of risks from ALDFG for these gear types as it is unclear what proportion of global effort by these gears occurs on FADs.

The study scope is on derelict fishing gear and excludes non-gear marine debris from fishing vessels, including intentionally-discarded types of debris that are specific to fishing vessels (e.g., bait boxes and plastic packing straps), and inadvertently leaked types of debris that are produced by all types of marine vessels (e.g., oil discharges during bunkering at sea, cargo nets, anti-fouling paint particles^69^).

Estimated ALDFG production rates for gillnet and trap gears were obtained from a fixed-effects meta-analysis by Richardson et al.^34^. We estimated ALDFG production rates for the other gears (Table S1). Mean ALDFG production rates and 95% highest posterior density intervals for these additional gear types were estimated using Bayesian generalized linear mixed regression models with Beta likelihood, except for pelagic longline and tuna purse seine, where a GLMM with zero-inflated Beta likelihood was used to account for zero values^38^. Additional and more recent records were compiled for some of these gears. For purse seine, trawl and seine net, Richardson et al.^34^ used a unit of percent of lost ‘net fragments’, which differed from and was not comparable to the rates used for other gear types. Richardson et al.^34^ aggregated some gears into high-level categories that we were able to disaggregate (pelagic and demersal longline, pelagic and bottom handline, non-tuna and tuna purse seine). ALDFG production rates for drifting and anchored FADs used by tuna purse seine and pole-and-line fisheries, spear, tong and rakes were not included in the meta-analysis by Richardson et al.^34^.

Gear-specific estimates of total catch from 2010 to 2014 were obtained from the Food and Agriculture Organization’s (FAO’s) *Discards Database for Global Marine Fisheries*^70^. Estimates of total catch for 2015 (the most current year available) were also obtained from Watson^71, 72^, which includes estimates for illegal, unregulated and unreported (IUU) catch and discards. Other available estimates of gear-specific global catch levels were explored but not used because of the use of aggregated gear categories. The sum of estimated gear- and fishery-specific geo-spatial area of fishing grounds for 2015 were obtained from Watson^71, 72^, who mapped landings to half-degree spatial cells. Gear-specific relative ecological and socioeconomic risks resulting from ALDFG were based on an assessment of six categories of adverse consequences summarized in Table 1. Table 1 describes factors that affect whether each adverse consequence occurs and the magnitude (size or severity) of the response, and defines explicit metrics against which each of the 18 fishing gears were assessed.

The overall gear-specific relative risk estimate was calculated as:

$$RR_{i} = R \, * \,E+ 2O$$

where *RR*_i_ is overall gear-specific relative risk of fishing gear *i*. *R* is the gear-specific ALDFG production (leakage) rate. *E* is an index of fishing effort of the quantity of fishing gear, calculated as the mean of annual gear-specific weight of catch *C* and gear-specific geospatial area of fishing grounds *A*. The product R*E is an index of gear-specific quantity of ALDFG. *O* is the gear-specific overall score of adverse ecological and socioeconomic outcomes per unit of ALDFG (Supplementary Material Section S3), assigned a weight of 2 to emphasize the adverse outcomes term to reduce the likelihood of false negatives where gears with high relative risk are assigned low *RR* scores due to gear quantity underestimates. For gear categories included in this study that did not have a direct match for *R*, *C* or *A*, Supplementary Material Section S2 describes the approaches that were employed. For *O*, an overall score was calculated as the mean of scores assigned to each of the six equally-weighted outcome categories. The values for *R*, *C*, *A* and *O* were normalized to a scale of 0 to 1, with 0 being no risk and 1 highest risk, as the ratio of the gear-specific value to the highest gear-specific value. Finally, the values for overall relative risk were also normalized to a scale of 0 to 1, with 0 being lowest relative risk and 1 being highest relative risk, using the following equation:

$$RR_{ni} = \frac{RRi-RRmin}{RRmax-RRmin}$$

where *RR*_*ni*_ is the normalized relative risk value of fishing gear *i*, *RRmin* is the minimum relative risk value, and *RRmax* the highest value.

## Supplementary Information

Supplementary Information.

## Data Availability

Correspondence and requests for materials should be addressed to E.G. All data used in the study are present in the paper, Supplementary Material, or cited publications.

## References

[1] Gilman E (2015). Status of international monitoring and management of abandoned, lost and discarded fishing gear and ghost fishing. Mar. Policy.

[2] FAO. *Voluntary Guidelines on the Marking of Fishing Gear* (Food and Agriculture Organization of the United Nations, 2019).

[3] Derraik J (2002). The pollution of the marine environment by plastic debris: A review. Mar. Pollut. Bull..

[4] Macfadyen, G., Huntington, T. & Cappel, R. *Abandoned, Lost or Otherwise Discarded Fishing Gear* (United Nations Environment Programme and Food and Agriculture Organization of the United Nations, 2009).

[5] Halpern B (2008). A global map of human impact on marine ecosystems. Science.

[6] Kroodsma D (2018). Tracking the global footprint of fisheries. Science.

[7] Thompson R (2004). Lost at sea: Where is all the plastic?. Science.

[8] Barnes D, Galgani F, Thompson R, Barlaz M (2009). Accumulation and fragmentation of plastic debris in global environments. Philos. Trans. R. Soc. B.

[9] Woodall L (2014). The deep sea is a major sink for microplastic debris. R. Soc. Open Sci..

[10] Jambeck J (2015). Plastic waste inputs from land into the ocean. Science.

[11] EUNOMIA. *Plastics in the Marine Environment* (Eunomia Research and Consulting, 2016).

[12] Geyer R, Jambeck J, Law K (2017). Production, use, and fate of all plastics ever made. Sci. Adv..

[13] Lebreton L (2017). River plastic emissions to the world’s oceans. Nat. Commun..

[14] Borrelle S (2020). Predicted growth in plastic waste exceeds efforts to mitigate plastic pollution. Science.

[15] Lebreton L (2018). Evidence that the Great Pacific Garbage Patch is rapidly accumulating plastic. Sci. Rep..

[16] Andrady A, Bergmann M, Gutow L, Klages M (2015). Persistence of plastic litter in the oceans. Marine Anthropogenic Litter.

[17] Kooi M, van Nes E, Scheffer M, Koelmans A (2017). Ups and downs in the ocean: Effects of biofouling on vertical transport of microplastics. Environ. Sci. Tech. Lib..

[18] Choy C (2019). The vertical distribution and biological transport of marine microplastics across the epipelagic and mesopelagic water column. Sci. Rep..

[19] Kane I (2020). Seafloor microplastic hotspots controlled by deep-sea circulation. Science.

[20] Galloway T, Cole M, Lewis C (2017). Interactions of microplastic debris through the marine ecosystem. Nat. Ecol. Evol..

[21] FAO. *The State of World Fisheries and Aquaculture 2020. Sustainability in Action* (Food and Agriculture Organization of the United Nations, 2020).

[22] Vegter A (2014). Global research priorities to mitigate plastic pollution impacts on marine wildlife. Endanger. Species Res..

[23] Scheld A, Bilkovic D, Havens D (2016). The dilemma of derelict gear. Sci. Rep..

[24] Hong S, Lee J, Lim S (2017). Navigational threats by derelict fishing gear to navy ships in the Korean seas. Mar. Pollut. Bull..

[25] Kiessling T, Gutow L, Thiel M, Bergmann M, Gutow L, Klages M (2015). Marine litter as habitat and dispersal vector. Marine Anthropogenic Litter.

[26] Angiolillo M, Fortibuoni T (2020). Impacts of marine litter on Mediterranean reef systems: From shallow to deep waters. Front. Mar. Sci..

[27] Hall M, Roman M (2013). Bycatch and Non-tuna Catch in the Tropical Tuna Purse Seine Fisheries of the World.

[28] UNGA. *Resolution A/RES/70/1, Transforming Our World: The 2030 Agenda for Sustainable Development* (United Nations General Assembly, 2015).

[29] Gilman, E., Chopin, F., Suuronen, P. & Kuemlangan, B. *Abandoned, Lost and Discarded Gillnets and Trammel Nets. Methods to Estimate Ghost Fishing Mortality, and Status of Regional Monitoring and Management* (FAO Fisheries and Aquaculture Technical Paper 600. ISBN 978-92-5-108917-0. Food and Agriculture Organization of the United Nations, 2016).

[30] National Academy of Sciences. Chapter 8—Marine Litter. In *Assessing Potential Ocean Pollutants: A Report of the Study Panel on Assessing Potential Ocean Pollutants to the Ocean Affairs Board*, National Academy of Sciences National Research Council Study Panel on Assessing Potential Ocean Pollutants (National Academy of Sciences, 1975).

[31] UNEP (2016). Marine Plastic Debris and Microplastics—Global Lessons and Research to Inspire Action and Guide Policy Change.

[32] FAO (2018). Our Oceans are Haunted. How “Ghost Fishing” is Devastating our Marine Environments.

[33] Greenpeace (2019). Ghost Gear: The Abandoned Fishing Nets Haunting Our Oceans.

[34] Richardson K, Hardesty B, Wilcox C (2019). Estimates of fishing gear loss rates at a global scale: A literature review and meta-analysis. Fish. Fish..

[35] Adam M, Jauhary A, Azheem M, Jaufer A (2019). Use of Anchored FADs in the Maldives—Notes for a Case Study for Assessing ALDFG.

[36] ISSF. *Non-Entangling and Biodegradable FADs Guide*. *Version 3* (International Seafood Sustainability Foundation, 2019).

[37] Evans D (2003). Hierarchy of evidence: A framework for ranking evidence evaluating healthcare interventions. J. Clin. Nurs..

[38] Liu F, Eugenio E (2018). A review and comparison of Bayesian and likelihood-based inferences in beta regression and zero-or-one-inflated beta regression. Stat. Methods Med. Res..

[39] Sutton A, Abrams K, Jones D, Sheldon T, Song F (2000). Methods for Meta-analysis in Medical Research.

[40] Marschke M, Berkes F (2005). Local level sustainability planning livelihoods: A Cambodian experience. Int. J. Sust. Dev. World.

[41] Banks R, Zaharia M (2020). Characterization of the Costs and Benefits Related to Lost and/or Abandoned Fish Aggregating Devices in the Western and Central Pacific Ocean.

[42] Gilman, E. *et al*. *Stakeholder Views on Methods to Identify Ownership and Track the Position of Drifting Fish Aggregating Devices Used by Tuna Purse Seine Fisheries with Reference to FAO's Draft Guidelines on the Marking of Fishing Gear. FAO Fisheries Circular 1163* (Food and Agriculture Organization of the United Nations, 2018).

[43] Shainee M, Leira B (2011). On the cause of premature FAD loss in the Maldives. Fish. Res..

[44] Widodo A, Wudianto X, Satria F (2016). Current status of the pole-and-line fishery in eastern part of Indonesia. Indonesian Fish. Res. J..

[45] Proctor, C. *et al*. *A Characterisation of FAD-Based Tuna Fisheries in Indonesian Waters*. ACIAR Project FIS/2009/059. ISBN:978-0-646-80326-5. (Australian Centre for International Agricultural Research, 2019).

[46] Sibisopere, M. The significant contribution of FADs to Solomon Taiyo Limited's fishing operations, in *Proceedings of Pêche Thonière et Dispositifs de Concentration de Poissons, Caribbean-Martinique, 15–19 Oct 1999*, IFREMER (Archive Institutionnelle de L’IFREMER, Sete, 2000).

[47] ISSF & IPNLF (2019). Skippers’ Guidebook to Pole-and-Line Fishing Best Practices.

[48] SPC (2019). Western and Central Pacific Fisheries Commission Tuna Fisheries Yearbook 2018.

[49] Bartram P, Kaneko J, Kucey-Nakamura K (2010). Sea turtle bycatch to fish catch ratios for differentiating Hawaii longline-caught seafood products. Mar. Policy.

[50] Hilborn R, Walters C (1992). Fish Stock Assessment: Choice, Dynamics and Uncertainty.

[51] Palomares M, Pauly D (2019). On the creeping increase of vessels’ fishing power. Ecol. Soc..

[52] Gilman E, Suuronen P, Chaloupka M (2017). Discards by global tuna fisheries. Mar. Ecol. Prog. Ser..

[53] ISSF (2019). A Snapshot of the Large-Scale Tropical Tuna Purse Seine Fishing Fleets as of June 2019.

[54] CMS (2011). Assessment of Bycatch in Gill Net Fisheries.

[55] Bell J, Watson R, Ye Y (2017). Global fishing capacity and fishing effort from 1950 to 2012. Fish. Fish..

[56] Rousseau Y, Watson R, Blanchard J, Fulton E (2019). Evolution of global marine fishing fleets and the response of fished resources. PNAS.

[57] Commission E (2020). Fleet Register.

[58] Taconet M, Kroodsma D, Fernandes J (2019). Global Atlas of AIS-based Fishing Activity—Challenges and Opportunities.

[59] Aldridge W (2018). A global mitigation hierarchy for nature conservation. Bioscience.

[60] MacMullen P (2003). Study to Identify, Quantify and Ameliorate the Impacts of Static Gear Lost at Sea.

[61] Gyi T (2020). Abandoned, Lost or Otherwise Discarded Fishing Gear (ALDFG) in Myanmar’s Myeik Archipelago.

[62] Suuronen P (2012). Low impact and fuel-efficient fishing—Looking beyond the horizon. Fish. Res..

[63] Gilman E, Passfield K, Nakamura K (2014). Performance of regional fisheries management organizations: Ecosystem-based governance of bycatch and discards. Fish Fish..

[64] Beverly, SD. Griffiths, D. & Lee, R. *Anchored Fish Aggregating Devices for Artisanal Fisheries in South and Southeast Asia: Benefits and Risks* (Food and Agriculture Organization of the United Nations, Regional Office for Asia and the Pacific, 2012).

[65] ICCAT. *Chair Report of the 1st Joint Tuna RFMO FAD Working Group Meeting* (Joint Tuna RFMO FAD Working Group, International Commission for the Conservation of Atlantic Tunas, 2017).

[66] Thai Union (2017). Marine Stewardship Council Pre-Assessment of the Pole and Line Fishery in Senegal.

[67] Miller K, Nadheeh I, Jauharee A, Anderson R, Adam M (2017). Bycatch in the Maldivian pole-and-line tuna fishery. PLoS ONE.

[68] Wudianto W, Satria F, Utama A (2017). Background Study on Gillnet Fisheries in Indonesian Waters.

[69] Galafassi S, Nizzetto L, Volta P (2019). Plastic sources: A survey across scientific and grey literature for their inventory and relative contribution to microplastics pollution in natural environments, with an emphasis on surface water. Sci. Total Environ..

[70] FAO, FAO Global Fisheries Discards Database, ISBN 978-92-5-131226-1, http://www.fao.org/fishery/static/TP633/ (Food and Agriculture Organization of the United Nations, 2019)*.*

[71] Watson R (2017). A database of global marine commercial, small-scale, illegal and unreported fisheries catch 1950–2014. Sci. Data.

[72] Watson, R. *Global Fisheries Landings. Version 4.0*, 10.25959/5c522cadbea37 (Institute for Marine and Antarctic Studies, University of Tasmania, 2019).

[73] Antonelis K, Huppert D, Velasquez D, June J (2011). Dungeness crab mortality due to lost traps and a cost-benefit analysis of trap removal in Washington State waters of the Salish Sea. N. Am. J. Fish. Manag..

[74] Bilkovic D, Havens K, Stanhope D, Angstadt K (2014). Derelict fishing gear in Chesapeake Bay, Virginia: Spatial patterns and implications for marine fauna. Mar. Pollut. Bull..

[75] Stelfox M, Hudgins J, Sweet M (2016). A review of ghost gear entanglement amongst marine mammals, reptiles and elasmobranchs. Mar. Pollut. Bull..

[76] Law K (2017). Plastics in the marine environment. Annu. Rev. Mar. Sci..

[77] Enrichetti F (2021). Fate of lost fishing gears: Experimental evidence of biofouling colonization patterns from the northwestern Mediterranean Sea. Environ. Pollut..

[78] Teuten E, Rowland S, Galloway T, Thompson R (2007). Potential for plastics to transport hydrophobic contaminants. Environ. Sci. Tech. Lib..

[79] Rochman C, Bergmann M, Gutow L, Klages M (2015). The complex mixture, fate and toxicity of chemicals associated with plastic debris in the marine environment. Marine Anthropogenic Litter.

[80] Chen Q (2018). Pollutants in plastics within the North Pacific Subtropical Gyre. Environ. Sci. Technol..

[81] Foley C, Feiner Z, Malinich T, Höök T (2018). A meta-analysis of the effects of exposure to microplastics on fish and aquatic invertebrates. Sci. Total Environ..

[82] Grade T (2019). Lead poisoning from ingestion of fishing gear: A review. Ambio.

[83] Seuront L, Nicastro K, McQuaid C, Zardi G (2020). Microplastic leachates induce species-specific trait strengthening in intertidal mussels. Ecol. Appl..

[84] Barnes D (2002). Biodiversity: Invasions by marine life on plastic debris. Nature.

[85] Masó M, Garcés J, Pagès F, Camp J (2003). Drifting plastic debris as a potential vector for dispersing Harmful Algal Blooms (HAB) species. Sci. Mar..

[86] Hallegraeff G, Hallegraeff G, Anderson D, Cembella A (2004). Harmful algal blooms: A global overview. Manual on Harmful Marine Microalgae.

[87] Erzini K (1997). An experimental study of gillnet and trammel net ‘ghost fishing’ off the Algarve (southern Portugal). Mar. Ecol. Prog. Ser..

[88] Bilkovic D, Havens K, Stanhope D, Angstadt L (2012). Use of fully biodegradable panels to reduce derelict pot threats to marine fauna. Conserv. Biol..

[89] English, E., Wagner, C. & Holmes, J. *The Effects of Marine Debris on Beach Recreation and Regional Economies in Four Coastal Communities: A Regional Pilot Study. Final Report* (Marine Debris Division, National Oceanic and Atmospheric Administration, 2019).

