# Supplementary material for: Highest risk abandoned, lost and discarded fishing gear
Source: Sci Rep. 2021 Mar 30;11:7195. doi: 10.1038/s41598-021-86123-3 (PMC8009918; doi:10.1038/s41598-021-86123-3)
Supplement: Supplementary file 1 — Supplementary Information. [file 41598_2021_86123_MOESM1_ESM.pdf]

Supplementary Material

Highest risk abandoned, lost and discarded fishing gears

Eric Gilman, Michael Musyl, Petri Suuronen, Milani Chaloupka, Saeid Gorgin, Jono Wilson, Brandon Kuczenski

Scientific Reports - 2021

Contents

S1. GEAR-SPECIFIC RATES OF PRODUCTION OF ALDFG 2

S2. ESTIMATES USED FOR GEAR-SPECIFIC ALDFG PRODUCTION RATE, ANNUAL CATCH AND AREA OF FISHING GROUNDS 7

S3. GEAR-SPECIFIC ADVERSE ECOLOGICAL AND SOCIOECONOMIC EFFECTS FROM ALDFG 8

S3.1. Ghost Fishing 9

S3.2. Distribution and transfer of toxins and microplastic into marine food webs 16

S3.3. Transporting invasive alien species and algae that cause red tides 22

S3.4. Habitat Alteration and Degradation 25

S3.5. Obstruction of In-use Fishing Gear, Navigation and Safety Risks at Sea 28

S3.6. Reduced Aesthetic and Use Value of Coastal and Nearshore Areas 34

S4. REFERENCES 38

S1. GEAR-SPECIFIC RATES OF PRODUCTION OF ALDFG

Table S1a summarizes estimated mean gear-specific rates of producing abandoned, lost and discarded fishing gear (ALDFG). Estimates were used from Richardson et al. (2019) for gillnet and trap (pot, fyke net, pound net). Table S1b provides the compiled, individual gear-specific rates of producing ALDFG that were used to estimate mean rates for the other gear types not derived from Richardson et al. (2019).

Table S1a. Mean gear-specific rates of abandonment, loss and discarding of fishing gear. Estimates from Richardson et al. (2019) are means and 95% confidence intervals (CIs) from a fixed-effects meta-analysis employing beta regression models. Estimates for other gears are posterior means with 95% highest posterior density intervals (HDIs), determined from Bayesian generalized linear mixed regression models (GLMMs) with Beta likelihood (Liu and Eugenio, 2018), except for pelagic longline and tuna purse seine where a GLMM with zero-inflated Beta likelihood was used to account for zero values (Gilman et al., 2020a). FAD=fish aggregating device. ALDFG=abandoned, lost and discarded fishing gear.

	ALDFG Rate			
**Gear**	**Mean**	**95% HDI and CI**	**Unit**	**Citation**
Drift gillnet	3.1	2.7 - 3.5	% lost	Richardson et al., 2019
Set and fixed gillnet	8.4	7.3 - 9.5	% lost	Richardson et al., 2019
Purse seine non-tuna	1.4	0.07 – 4.6	% lost	Natural Resources Consultants, 1990; Deshpande et al., 2020
Purse seine tuna (excluding FADs)	8.1	0.0 – 51.0	% lost	Natural Resources Consultants, 1990; IUCN, 2019
Beach seine	4.5	0.02 – 21.0	% lost	Natural Resources Consultants, 1990
Boat seine	5.1	0.3 – 16.8	% lost	Natural Resources Consultants, 1990; UNEP MAP, 2015; Deshpande et al., 2020
Midwater trawl	10.5	0.02 – 43.4	% lost	Natural Resources Consultants, 1990
Bottom trawl	3.5	1.9 – 5.3	% lost	Natural Resources Consultants, 1990; Deshpande et al., 2020
Pot	19.0	18.9 – 20.0	% lost	Richardson et al., 2019
Fyke net	4.1	3.8 - 4.5	% lost	Richardson et al., 2019
Pound net	2.6	2.4 - 2.8	% lost	Richardson et al., 2019
Drifting FADs used by tuna purse seine	47.9	19.0 – 78.0	% abandoned, lost and discarded	Maufroy et al., 2015; Gilman et al., 2018; Banks and Zaharia, 2020
Anchored FADs used by tuna purse seine	81.0	NA	% lost	MacFadyen et al., 2009
Anchored FADs used by pole-and-line	20.0	NA	% lost	Adam et al., 2019
Anchored FADs used by dolphinfish and greater amberjack purse seine	>76.0	NA	% lost	Lleonart et al., 1999; Sinopoli et al., 2020

Pelagic longline	2.4	0.01 – 8.9	% lost	Anderson and Waheed 1990; Natural Resources Consultants, 1990; Gilman et al., 2018; DOS, 2020
Demersal longline	18.7	5.7 – 34.7	% lost	Natural Resources Consultants, 1990; Glass et al., 2000; Tasliel, 2008; Ayaz et al., 2010; Webber and Parker, 2012; Yildiz and Karakulak, 2016
Handline, surface, midwater and bottom	21.3	4.1 – 44.3	% lost	Natural Resources Consultants, 1990; Rouxel, 2017
Troll	3.5	0.1 – 13.9	% lost	Natural Resources Consultants, 1990
Pole-and-line (excluding FADs)	20.9	0.9 – 56.6	% lost	Natural Resources Consultants, 1990
Rakes, tongs	0.01	NA	% lost	Natural Resources Consultants, 1990
Speargun	0.04	NA	% lost	Frisch et al., 2008

Table S1b. Gear-specific rates of producing ALDFG.

Gear	ALDFG Rate		Citation
	Value	Unit	
**Fish Aggregating Devices (FADs)**			
Drifting FADs used by tuna purse seine	25.3[a]	% of deployed that are lost and abandoned	Maufroy et al., 2015
Drifting FADs used by tuna purse seine	3.7	% of deployed that ran aground (lower-bound estimate of % that are abandoned, lost and discarded) – an earlier instance of the same dataset used by Banks and Zaharia, 2020 so exclude from overall estimate	Escalle et al., 2017
Drifting FADs used by tuna purse seine	21.4[b]	% that are lost (lower-bound estimate of % that are abandoned, lost and discarded)	Gilman et al., 2018
Drifting FADs used by tuna purse seine	86.8	% of deployed not retrieved and thus abandoned and lost (lower-bound estimate of % that are abandoned, lost and discarded)	Banks and Zaharia, 2020
Anchored FADs used by tuna purse seine	81	% lost	MacFadyen et al., 2009
Anchored FADs used by tuna pole-and-line	82[3]	% lost per year	Shainee and Leira, 2011
Anchored FADs used by tuna pole-and-line	20[c]	% lost per year	Adam et al., 2019
Anchored FADs used by dolphinfish and greater amberjack purse seine	>76	% lost per year	Lleonart et al., 1999; Sinopoli et al., 2020

**Pelagic longline**			
Pelagic longline	0	% discarded per set	DOS, 2020
Pelagic longline	0.6	% of branchline terminal tackle (hook and section of line) lost due to bite-offs	Gilman et al., 2018
Pelagic longline	3	% of branchline terminal tackle (hook and section of line) lost due to bite-offs	Anderson and Waheed 1990
Pelagic longline (US north Atlantic)	0.1	% loss per year	Natural Resources Consultants, 1990
Pelagic longline (US south Atlantic)	0.1	% loss per year	Natural Resources Consultants, 1990
Pelagic longline (US Gulf of Mexico and Caribbean)	0.1	% loss per year	Natural Resources Consultants, 1990
**Demersal longline**			
Demersal longline	79.2	% loss	Ayaz et al., 2010
Demersal longline	14.5	% loss	Yildiz and Karakulak, 2016
Demersal longline	4.7	% loss	Webber and Parker, 2012
Demersal longline	13.5	% loss of hooks	Glass et al., 2000
Demersal longline (US south Atlantic)	0.1	% loss per year	Natural Resources Consultants, 1990
Demersal longline (trotline) (US south Atlantic)	0.1	% loss per year	Natural Resources Consultants, 1990
Demersal longline (US Gulf of Mexico and Caribbean)	0.1	% loss per year	Natural Resources Consultants, 1990
Demersal longline (trotline) (US Gulf of Mexico and Caribbean)	0.1	% loss per year	Natural Resources Consultants, 1990
Demersal longline (US west coast)	21	% loss per year	Natural Resources Consultants, 1990
Demersal longline	932	km of line lost per season	Tasliel, 2008
**Longline not specified**			
Longline (pelagic or demersal not specified)	28.6	% loss of length of line with hooks	UNEP MAP, 2015
Longline (pelagic or demersal not specified)	4.4	% loss of total gear owned	Deshpande et al., 2020
**Pole-and-line - excluding fish aggregating devices**			
Pole-and-line (California)	20	% loss per year	Natural Resources Consultants, 1990
Pole-and-line (US Caribbean)	1	% loss per year	Natural Resources Consultants, 1990

**Purse seine, tuna - excluding fish aggregating devices**			
Purse seine, tuna (US north Atlantic region)	0.1	% loss per year	Natural Resources Consultants, 1990
Purse seine, tuna	0.0	% loss per year	IUCN, 2019
**Purse seine, non-tuna**			
Purse seine, non-tuna	0.4	% loss of total gear owned	Deshpande et al., 2020
Purse seine, non-tuna (US south Atlantic region)	0.1	% loss per year	Natural Resources Consultants, 1990
Purse seine, non-tuna (US Gulf of Mexico and Caribbean region)	0.1	% loss per year	Natural Resources Consultants, 1990
Purse seine, non-tuna (US west coast and Alaska region)	0.51	% loss per year	Natural Resources Consultants, 1990
Purse seine, non-tuna (US north Atlantic region)	0.1	% loss per year	Natural Resources Consultants, 1990
**Bottom handline**			
Bottom handline	5.3[d]	% loss of hooks	Rouxel, 2017
Bottom handline (US north Atlantic)	0.1	% loss per year	Natural Resources Consultants, 1990
Bottom handline (US south Atlantic)	0.1	% loss per year	Natural Resources Consultants, 1990
Bottom handline (US Hawaii, Am. Samoa, Guam, Northern Mariana Is.)	50.0	% loss per year	Natural Resources Consultants, 1990
**Surface and midwater handline**			
Handline tuna (US Hawaii, Am. Samoa, Guam, Northern Mariana Is.)	35.0	% loss per year	Natural Resources Consultants, 1990
**Troll**			
Troll (US north Atlantic)	0.1	% loss per year	Natural Resources Consultants, 1990
Troll (US south Atlantic)	0.1	% loss per year	Natural Resources Consultants, 1990
Troll (US Gulf of Mexico and Caribbean)	0.6	% loss per year	Natural Resources Consultants, 1990
Troll (US west coast)	1.9	% loss per year	Natural Resources Consultants, 1990

**Midwater trawl**			
Midwater trawl (US north Atlantic region)	0.1	% loss per year	Natural Resources Consultants, 1990
Midwater trawl (US west coast and Alaska region)	1.0	% loss per year	Natural Resources Consultants, 1990
**Bottom trawl**			
Bottom trawl	3.1	% loss of total gear owned	Deshpande et al., 2020
Bottom trawl (US south Atlantic region)	2.7	% loss per year	Natural Resources Consultants, 1990
Bottom trawl (US Gulf of Mexico and Caribbean region)	2.7	% loss per year	Natural Resources Consultants, 1990
Bottom trawl (US west coast and Alaska region)	3.5	% loss per year	Natural Resources Consultants, 1990
Bottom trawl (US north Atlantic region)	3.0	% loss per year	Natural Resources Consultants, 1990
**Trawl**, not specified	25.0	% loss of length of net excluding codend	UNEP MAP, 2015
**Beach seine**			
Beach seine (US south Atlantic region)	0.01	% loss per year	Natural Resources Consultants, 1990
Beach seine (US Gulf of Mexico and Caribbean region)	0.01	% loss per year	Natural Resources Consultants, 1990
Beach seine (US west coast and Alaska region)	0.01	% loss per year	Natural Resources Consultants, 1990
Beach seine (US north Atlantic region)	0.04	% loss per year	Natural Resources Consultants, 1990
**Boat seine**			
Boat seine	6.4	% loss of length of net	UNEP MAP, 2015
Boat seine	1.8	% loss of total gear owned	Deshpande et al., 2020
Boat seine (US north Atlantic region)	0.1	% loss per year	Natural Resources Consultants, 1990
Boat seine (US south Atlantic region)	0.1	% loss per year	Natural Resources Consultants, 1990
Boat seine (US Gulf of Mexico and Caribbean region)	0.1	% loss per year	Natural Resources Consultants, 1990
**Rakes, tongs**	0.01	% lost per year	Natural Resources Consultants, 1990

**Speargun**	0.04[e]	% lost per deployment	Frisch et al., 2008

[a] Calculated as the sum of (a) 9.9% of deployed that ran aground - a lower-bound estimate of % that are lost and abandoned, and (b) 24.4% of deployed that drift outside of fishing grounds and are lost or abandoned. The authors estimated that 57% of deployed that drifted outside of tuna purse seine fishing grounds at least once during their at-sea period but some portion of these FADs may re-enter the fishing grounds or enter fishing grounds of other tuna purse seine fleets – so this is an upper bound estimate of % lost and abandoned and not retrieved. However, the authors also report that 67.7% of dFADs were outside of French fishing grounds 100% of the time, of which 72.1% were outside of all tuna purse seine fishing grounds, such that 48.8% of dFADs were outside of the fishing grounds. Some of these may be recovered or used by artisanal fisheries. We roughly estimate that half of these 48.8% of dFADs are permanently lost and abandoned.

[b] ±2.8 SE, N=50.

[c] Estimates are both for the Maldives anchored FAD network for the tuna pole-and-line fishery, from different time periods.

[d] 16% chance that 1 of 3 of hooks will be lost per day.

[e] Estimated loss rate based on 3 fishing gear items (2 spear tips, 1 gun rubber) lost per 70 deployments of vessel anchor (hangs), if each lost item is ca. 1% of the total fishing gear volume, results in a loss rate of 0.043% per hang.

S2. ESTIMATES USED FOR GEAR-SPECIFIC ALDFG PRODUCTION RATE, ANNUAL CATCH AND AREA OF FISHING GROUNDS

For fishing gear categories included in the study that did not have a direct gear category match for ALDFG production rates from Table S1, the following approach was used:

- For barrier, fence, weir, corral, the Table S1 value for pound net was used.
- For hand dredge, harpoon, spear, lance, tongs, rakes, the mean of Table S1 values for spearfishing and rakes/tongs was used.
- For pole-and-line including anchored FADs, the mean of Table S1 values for pole-and-line and anchored FADs used by pole-and-line was used.
- For tuna purse seine including FADs, the mean of the Table S1 purse seine tuna value and half of the value for drifting FADs used by purse seine tuna was used, because, explained in the methods section, about half of the catch from global tuna purse seine fisheries is from sets on drifting FADs and because anchored FAD use in tuna purse seine fisheries was assumed to be small relative to drifting FAD use (Miller et al., 2016; ICCAT, 2017; Gilman et al., 2018; Banks and Zaharia, 2020).

Similarly, for gear categories included in the study that did not have a direct gear category match for global annual catch levels from Watson (2017) and Gilman et al. (2020), the following approach was used:

- For (a) fyke net, (b) pound net and (c) barrier, fence, weir, corral, the mean of (i) one third of the estimate from Gilman et al. (2020) for barrier, fence, trap, weir, and (ii) one fourth of the estimate from Watson (2017) for traps was used.
- For pot, the mean of (i) Gilman et al. (2020) estimate for pot, and (ii) one fourth of the estimate from Watson (2017) for trap was used.
- For drift gillnet, the mean of (i) the sum of the Gilman et al. (2020) estimate for gear category drift gillnet and one quarter of the estimate for gear category drift and anchored gillnet, and (ii) a quarter of the Watson (2017) estimate for the gear category gillnet, trammel net, combination gillnet-trammel net was used.
- For set and fixed gillnet, trammel net, combination gillnet/trammel net, the mean of (i) the sum of the Gilman et al. (2020) estimate for gear category anchored gillnet, estimate for

trammel net, and three quarters of the estimate for gear category drift and anchored gillnet, and (ii) three quarters of the Watson (2017) estimate for the gear category gillnet, trammel net, combination gillnet-trammel net was used.

- For demersal longline, the mean of (i) the sum of Gilman et al. (2020) estimate for demersal longline and half of the estimate for demersal and pelagic longline, and (ii) Watson (2017) estimate for demersal longline was used.
- For pelagic longline, the mean of (i) the sum of Gilman et al. (2020) estimate for pelagic longline and half of the estimate for demersal and pelagic longline, and (ii) Watson (2017) estimate for pelagic longline was used.
- For beach seine, the mean of (i) the estimate of Gilman et al. (2020) for beach seine. and (ii) the sum of the Watson (2017) estimate for beach seine and half of the estimate for seine net, vessel or beach not specified was used.
- For boat seine, the mean of (i) the estimate of Gilman et al. (2020) for boat seine. and (ii) the sum of the Watson (2017) estimate for vessel seine and half of the estimate for seine net, vessel or beach not specified was used.

And, similarly, for gear categories included in the study that did not have a direct gear category match for the geo-spatial area of fishing grounds from Watson (2017, 2019), the following approach was used:

- For barrier, fence, corral; fyke net; pot and pound net, the Watson (2017, 2019) estimate for trap was used.
- For set and fixed gillnet, trammel net, combination gillnet/trammel net, the Watson (2017, 2019) sum of estimates for gillnet and trammel net and for gillnet, trammel net, combination gillnet-trammel net was used.
- For drift gillnet, 20% of the Watson (2017, 2019) value for set and fixed gillnet, trammel net, and combination gillnet/trammel net was used.
- For hand dredge, harpoon, spear, lance, tongs, rakes, hand-collected, the Watson (2017, 2019) sum of estimates for hand dredge; harpoon, spear, lance; tongs; raking device; and hand-collected was used.
- For (a) handline, (b) pole-and-line and (c) troll, 75% of the Watson (2017, 2019) estimate for handline, pole-and-line, troll, jig was used.
- For beach seine, the Watson (2017, 2019) sum of estimates for beach seine and half of the estimate for seine net (vessel or beach not specified) was used.
- For boat seine, the Watson (2017, 2019) sum of estimates for vessel seine and half of the estimate for seine net (vessel or beach not specified) was used.

S3. GEAR-SPECIFIC ADVERSE ECOLOGICAL AND SOCIOECONOMIC EFFECTS FROM ALDFG

The following subsections assess six categories of adverse effects that can result from ALDFG from 18 different gear types, for a single unit of derelict gear that is typically produced by that gear – e.g., a pot or string of pots, a section of small-mesh polyamide or polyester webbing from a small pelagics purse seine net. Metrics for assessment against each criterion are from Table 1. Each criterion was assigned a score on a scale of 0 to 1, with 0 being no risk, and 1 being highest possible risk, identified in parentheses in the rationale column of each of the tables included in this section. Within each of the 6 categories, an overall gear-specific score is the mean of the scores assigned to each criterion, where criteria within each category are weighted equally. The gear boat seine includes Danish seine (also called anchor seining) and Scottish seine (also called fly-dragging).

S3.1. Ghost Fishing
Table S2 assesses gear-specific relative risks from ghost fishing, including ingestion of components of ALDFG. From Table 1, the following three metrics were assessed to determine gear-specific relative risks:

(1) Risk of ghost fishing mortality when gear initially becomes derelict;
(2) Duration of ghost fishing efficiency, accounting for effects of self-baiting, prevalence of use of designs intended to reduce ghost fishing efficiency, environmental conditions that could disable the gear, exposure to vessels and in-use mobile fishing gear, local abundance of species susceptible to capture; and
(3) Vulnerability and socioeconomic value of species susceptible to ghost fishing, including ingestion of components of ALDFG.

For the second criterion, if the duration of ghost fishing efficiency typically occurs longer than 1 year, then 1.0 is assigned for that criterion.

Table S2. Gear-specific relative risk of ghost fishing and ingestion of ALDFG.

Gear	Rationale	Citations
Barrier, fence, weir, corral	1. Abandoned barriers, fences, weirs and corrals would initially have the same fishing efficiency, and risk of ingestion of gear components, as in-use gear. Derelict synthetic net fragments may also cause ghost fishing. A typical unit of derelict gear might be a lost gear component or an entire abandoned trap. These traps are relatively permanent structures, fixed in place and thus have a low risk of becoming lost, except during rare, strong storm events. (1.0)<br><br>2. While traps made of natural, biodegradable materials likely lose their fishing efficiency rapidly following abandonment, ALDFG from fisheries using synthetic netting and frames will have a duration of ghost fishing efficiency that may exceed 1 year. We conservatively estimate that, on average, the duration of ghost fishing efficiency is > 1 year. (1.0)<br><br>3. Assumed to be the same as pots. (1.0)	FAO, 2001; Slack-Smith, 2001; Macfadyen et al., 2009; Samoilys et al., 2011; Fitri and Pramonowibowo, 2015
Fyke net	1. Derelict fyke nets, when initially lost or abandoned, would retain some or all of their in-use fishing efficiency, depending on the position and structure of the trap. (1.0)<br><br>2. While fyke nets made of natural, biodegradable materials likely lose their fishing efficiency rapidly following loss and abandonment, ALDFG from fyke net fisheries using synthetic netting and frames will have a duration of ghost fishing efficiency that may exceed 1 year. We conservatively estimate that, on average, the duration of ghost fishing efficiency is > 1 year. However, the fishing efficiency of the trap will decline as the position is altered and structure of the net changes over time. (1.0)<br><br>3. Assumed to be the same as pots. (1.0)	FAO, 2001; Samoilys et al., 2011
Pot	1. Derelict pots, when initially lost or abandoned, would likely retain the same fishing efficiency as in-use gear. (1.0)	High and Worlund, 1979; Breen, 1989; Jones, 1995; Stevens et al., 2000; Bullimore et al., 2001; Al-

	2. Some derelict pots can maintain ghost fishing efficiency for years. The duration of ghost fishing efficiency depends in part on the conditions where the gear ends up (see set and fixed gillnets and trammel nets, below). There is a high risk of self-baiting in derelict pots. In some pot fisheries, pots are made of natural and degradable materials, and in some fisheries, pots made of synthetic materials may use degradable escape panels and cords, but it is assumed that this applies to a small proportion of global pot fisheries. (1.0)<br><br>3. Derelict pots can result in substantial ghost fishing mortality of marketable species, including target species, and of vulnerable bycatch species. (1.0)	Masroori et al., 2004; Havens et al., 2008; Giordano et al., 2010; Antonelis et al., 2011; Samoilys et al., 2011; Voss et al., 2012; Matthews et al., 2012; Maselko et al., 2013; Arthur et al., 2014; Uhrin et al., 2014; Bilkovic et al., 2014; Renchen et al., 2014; Stevens, 2014; Scheld et al., 2016; DelBene et al., 2019; Sukhsangchan et al., 2020
Pound net	Assumed to have similar ghost fishing consequences as derelict barriers, fences, weirs and corrals.	Cheng and Chen, 1997; FAO, 2001; Gilman, 2009; Gilman et al., 2010; Silva et al., 2011
Gillnet, drift	1. Lost and abandoned driftnets can initially have the same fishing efficiency as in-use gear. Discarded webbing might have some fishing efficiency depending on the habitat type and structure where it settles to the seabed. (1.0)<br><br>2. While lost and abandoned driftnets might take years before sinking or running aground, they would retain some fishing efficiency for up to a few months before collapsing into a floating mass of netting, and then might still retain a small degree of fishing efficiency. Once it sinks or runs aground, whether or not and for how long the driftnet retains ghost fishing efficiency depends in part on the conditions where the derelict gear ends up (see set and fixed gillnets and trammel nets, below). Monofilament and multi-monofilament polyamide are typically used for gillnet and trammel net webbing, and fragments of this netting sink. (0.5)<br><br>3. Both vulnerable species (e.g., marine turtles, sharks, rays, marine mammals, seabirds) and principal market species are susceptible to ghost fishing mortality. Once the driftnet sinks or runs aground, the target species are no longer susceptible to capture, but other marketable species might have ghost fishing catch risk. (1.0)	Eisenbud, 1985; Gerrodette et al., 1987; Breen, 1990; Mio et al., 1990; Jones, 1995; Donohue et al., 2001; Gilardi et al., 2010; Wilcox et al., 2014; Gilman et al., 2016; King Net, 2020
Gillnet, set and fixed; trammel net; combination gillnet/trammel net	1. Anchored and staked gillnets, trammel nets and combination gillnets/trammel nets can, under certain circumstances, have an initial ghost fishing efficiency that is the same or close to that of in-use gear when abandoned and lost. If set (anchored) gillnets, which can be designed to fish anywhere from the surface, midwater to at or near the seabed, break from anchor lines, it might float and drift like a lost drift gillnet. Made of polyamide, which sinks, discarded webbing might have some fishing efficiency depending on the habitat type and structure where it occurs on the seabed. (1.0)	Jones, 1995; Erzini et al., 1997; FAO, 2001; MacMullen et al., 2003; Revill and Dunlin, 2003; Baeta et al., 2009; Gilardi et al., 2010; Gilman et al., 2016; Deshpande et al., 2020

	2. Derelict set and fixed gillnets, trammel nets and combination gillnets/trammel nets can retain some ghost fishing efficiency for several years under certain conditions, such as in deep, cold waters. There is a high risk of self-baiting in derelict gillnets, in some cases over several years. The long duration of ghost fishing efficiency includes gear that was set for fishing prior to becoming derelict (lost or abandoned), and given certain conditions of the fishing grounds, including:<br>• Substrate is protected and with 3-dimensional features on which the gear can entangle, and not open and flat<br>• High local abundance of organisms susceptible to capture in the gear<br>• Relatively low local abundance of biofouling organisms, debris and particulate matter<br>• Relatively deep, with a low degree of exposure to environmental forces (wave energy, storms, currents) that can disable the derelict gear<br>• Located at a site where it is unlikely to be disabled by passing vessels and mobile fishing gear (e.g., bottom trawls, dredges)<br>(1.0)<br><br>3. Both vulnerable species and principal market species are susceptible to ghost fishing mortality. Once the gillnet/trammel net loses its profile, target species may no longer be susceptible to capture, but other marketable species might have ghost fishing catch risk. (1.0)	
Hand dredge, harpoon, spear, lance, tongs, rakes, hand-collected (including diving) - shore- and boat-based	1, 2, and 3. There is no risk of ghost fishing by these gears. If abandoned, lost or discarded, these active gears would have no fishing efficiency. (0.0)	Barnette, 2001; FAO, 2001; Bjordal, 2002; Frisch et al., 2008; Karnchanawong and Limpiteeprakan, 2009; James et al., 2018; ODFW, 2019
Handline, midwater, surface and bottom	1. When it becomes abandoned, lost or discarded, monofilament nylon lines and hooks would sink to the seabed. Derelict floats, marker buoys and lines, if used, would pose minimal ghost fishing risk. Derelict handline gear poses a relatively low risk of ghost fishing, from entanglement in line and ingestion of catch along with terminal tackle. There is likely minimal risk of ghost fishing by ALDFG from handline gear that sink to the seabed. Anchored FADs are used by some handline fisheries, but likely a small proportion globally. (0.0)<br><br>2. The risk of entanglement in derelict handline gear would likely rapidly decline if the line and terminal tackle becomes incorporated into hard substrate or buried in sediment, but line entangled on a three-dimensional surface could retain ghost fishing until the monofilament line degrades past some threshold, which may be > 1 year. (0.1)<br><br>3. Principal market species are not susceptible to capture in derelict handline gear. ALDFG from commercial handline gear can entangle wildlife and hooked fish and lead sinkers can be ingested. For example, a harbor seal died of lead toxicosis following ingestion of a lead sinker possibly from a	de San and Pages, 1998; Desurmont and Chapman, 2000; Zabka et al., 2006; Campagna et al. 2007; Macusi et al., 2015; Widodo et al., 2016

	California commercial rockfish hook-and-line fishery. And, for example, southern elephant seals have been observed entangled in line from a squid jig handline fishery, which may have been discarded. However, given that derelict handline gear sinks, the risk of vulnerable species ghost fishing is relatively low. (0.1)	
Longline, demersal	1. Lost and abandoned demersal longlines would initially have the same fishing efficiency as in-use gear. (1.0)<br><br>2. Abandoned and lost demersal longlines would quickly, perhaps within a few days, lose their fishing efficiency once hooks are no longer baited – once baits fall from hooks due to degradation of tissues (depends on temperature), mechanical action, are depredated, degrade or captured fish escape, are depredated or degrade. (0.0)<br><br>3. Target and vulnerable bycatch species would be susceptible to capture, as with in-use gear, but only for a short duration while baited hooks remained available. Discarding spent bait retaining hooks can injure and kill seabirds; however, this is likely a rare occurrence because (i) hooks tend to be permanently affixed to branchlines, and (ii) in some fisheries the value of hooks is perceived to be high enough to result in fishers taking the time and safety risk to remove terminal tackle from unwanted sharks and other catch before discarding. Discarded gear components (mainline, snoods, hooks) would sink to the seabed and have no fishing efficiency. (0.0)	Natural Resources Consultants, 1990; Webber and Parker, 2012; Galbraith et al., 2014
Longline, pelagic	1. Lost and abandoned pelagic longlines would initially have the same fishing efficiency of in-use gear. In small scale pelagic longline fisheries that do not use radio buoys or other electronic technology to track the location of the gear while fishing and drifting, the entire mainline or sections of mainline may be lost, especially when there are strong currents. Illegal gear may be abandoned when there is a risk of detection. In industrial longline fisheries, loss of an entire set or section of mainline is very rare, which might occur, for example, when a baleen whale entangles with and swims off with a section of the mainline; where instead discarding sections of damaged monofilament line, and bite-offs of terminal tackle, are likely main sources of ALDFG. (1.0)<br><br>2. As with demersal longlines, the ghost fishing efficiency of lost and abandoned pelagic gear would be lost quickly, perhaps within a few days, once hooks are no longer baited or contain catch. (0.0)<br><br>3. Target and vulnerable bycatch species would be susceptible to capture, as with in-use gear, but only briefly while baited hooks remained available. See demersal longline for risks of seabird ghost fishing from discarded spent bait retaining hooks. Otherwise, discarded gear components would sink to the seabed and have no fishing efficiency. (0.0)	Anderson and Waheed 1990; Natural Resources Consultants, 1990; Jones, 1995; Laist, 1997; Macfadyen et al., 2009; Gilman et al., 2018; Save The Med Foundation, 2020
Pole-and-line, including anchored FADs	1. Derelict terminal tackle from pole-and-line gear will sink and likely has minimal ghost fishing efficiency. As with handlines, derelict line and terminal tackle from pole-and-line gear may pose a risk of ghost fishing from entanglement in line, and ingestion of catch along with terminal tackle, but the risk of ghost fishing is relatively low. There is likely very low risk of ghost fishing by ALDFG from pole-and-line gear that sink to the seabed in deep waters.<br>As with derelict drifting FADs using conventional entangling designs (see tuna purse seine), in some pole-and-line fisheries, derelict anchored FADs (called rumpons in Indonesia and payaos in the	Natural Resources Consultants, 1990; de San and Pages, 1998; Desurmont and Chapman, 2000; Sibisopere, 2000; Shainee and Leira, 2011; Widodo et al., 2016; Miller

	Philippines) may use netting for the subsurface appendage under a raft and may incorporate twine into the raft, which can result in ghost fishing of sharks, marine turtles and other species, and the float line and lines connecting floats to rafts may result in ghost fishing mortalities – documented to occur for instance for loggerhead sea turtles. In some regions, anchored FADs are designed without netting, where the surface structure and appendage are made from floats, barrels, plastic bottles, plastic strapping, plastic sheets, polystyrene slabs, bamboo, palm fronds, and various other natural and synthetic materials connected with lines, which very likely pose a lower risk of ghost fishing than designs with appendages and float with webbing. And some pole-and-line anchored FADs that use a buoy or drums connected by a floatline to anchors likely have very low ghost fishing efficiency. For example, anchored FADs used in Indonesian tuna fisheries use synthetic lines, where the line connecting the surface structure to cement blocks can be several km in length; the surface structure may be made of foam encased with car tires, steel cylinders, or bamboo; and the appendage is typically constructed of palm fronds or other natural, biodegradable materials, with no webbing.<br>A majority of the catch from pole-and-line fisheries comes from fisheries that fish on anchored as well as drifting FADs: The Maldives and Indonesia pole-and-line fisheries, which supply over half of global pole-and-line catch, use anchored FADs, and several other smaller pole-and-line fisheries (e.g., Japan Okinawa, Solomon Islands) also make use of anchored FADs. Not all derelict anchored FAD designs pose a risk of ghost fishing. The number of FADs per pole-and-line vessel is likely relatively smaller than the ratio for tuna purse seine vessels. (0.25)<br><br>2. The risk of entanglement in derelict line and terminal tackle would likely rapidly decline if it becomes incorporated into hard substrate or buried in sediment, but line entangled on a three-dimensional surface could retain ghost fishing until the monofilament line degrades beyond some a threshold, which may be > 1 year. Risk of entanglement in derelict anchored FAD netting, if used, and lines would persist for > 1 year. (0.6)<br><br>3. Principal market species are not susceptible to capture in derelict pole-and-line gear. Discussed above, sharks, sea turtles, marine mammals and other vulnerable species may be susceptible to capture in some designs of derelict anchored FADs using netting as has been observed in drifting FADs using conventional entangling as well as less-entangling (sausages of netting) designs. Lines used in anchored FADs also pose an entanglement risk. (0.25)	et al., 2016, 2017; Thai Union, 2017; Defaux et al., 2018; Adam et al., 2019; ISSF and IPNLF, 2019; Proctor et al., 2019
Purse seine, non-tuna	1. In large purse seines, the loss of a full net is a very rare event. Sections of the net, however, may be ripped off when the net entangles in the purse cable or in another section of the net. This type of event is also infrequent – purse seiners avoid contact with the seabed because the small mesh netting can be easily damaged. Non-tuna (anchovy, capelin, herring, sardine, salmon, etc.) purse seine nets are typically made of polyester and polyamide, and fragments of this netting sink. Net fragments that occur on the seabed have minimal ghost fishing efficiency. (0.1)<br><br>2. Net fragments that occur on the seabed likely quickly (< 1 year) become embedded and lose whatever small ghost fishing efficiency that they originally had. (0.0)	Prado, 1990; Bjordal, 2002; Gilman et al., 2013; Galbraith et al., 2014; Tang et al., 2018; Zhou et al., 2019; Deshpande et al., 2020; King Net, 2020; Netmark, 2020

	3. Purse seine net fragments that have sunk to the seabed have no catch risk for target species and likely have very low catch risk for a short duration for vulnerable species. (0.1)	
Purse seine, tuna, including drifting and anchored FADs	1. In large purse seines, such as used by tuna purse seiners, the loss of an entire net is a very rare event. Sections of the net, however, may be ripped off when the net entangles in the purse cable, the prop, or in another section of the net. This type of event is also extremely infrequent, perhaps one event of a net fragment loss per year per vessel. Tuna purse seine webbing is typically made of polyamide and polyester, and fragments of this netting sinks (however, see Stelfox et al. [2020] who reported observing drifting derelict net fragments from tuna purse seine fisheries). Net fragments that occur on the seabed have minimal ghost fishing efficiency. Derelict FADs may have high ghost fishing efficiency. About half of the catch by global tuna purse seine fisheries is from FAD sets. Caps have recently been adopted on the number of active drifting FADs per vessel. There has been a recent transition to the use of less- and non-entangling designs of drifting FADs, which reduces the ghost fishing efficiency of this fishing gear component – three of the four tuna RFMOs now require tuna purse seine vessels to use only non-entangling designs (WCPFC plans to consider adopting a non-entangling FAD requirement at their December 2020 annual session of the Commission). (0.5)<br><br>2. Fragments of tuna purse seine nets that occur on the seabed likely quickly become embedded and lose whatever small ghost fishing efficiency that they originally had. Derelict FADs may maintain ghost fishing efficiency for > 1 year, until the FAD sinks or runs aground. (0.8)<br><br>3. Purse seine net fragments that have sunk to the seabed have no catch risk for target species and likely very low catch risk for vulnerable species.<br>The subsurface appendage and netting on rafts of derelict drifting FADs using conventional 'entangling' and 'less-entangling' designs can entangle silky and oceanic whitetip sharks, olive Ridley sea turtles, porpoises, and other species (but not likely market species). However, discussed above, there has been a recent transition to the use of less- and non-entangling designs of drifting FADs. As with derelict drifting FADs using conventional entangling designs, derelict anchored FADs may use netting for the subsurface appendage under a raft and may incorporate twine into the raft, which can result in ghost fishing of sharks, marine turtles and other species, and the float line and lines connecting floats to rafts may have result in ghost fishing mortalities – documented to occur for instance for loggerhead sea turtles. (0.5)	Prado, 1990; Bjordal, 2002; Itano, 2002; Chanrachkij and Loog-on, 2003; Filmalter et al., 2013; Gilman et al., 2013; Stelfox et al., 2014; Balderson and Martin, 2015; Blasi et al., 2016; ICCAT, 2017; Gilman et al., 2018; ISSF, 2019; WCPFC, 2019; Zhou et al., 2019; Atlantic Avitaillement, 2020
Seine, beach	1. This active fishing gear, which can be shore- or boat-based, has a very low risk of being lost or abandoned. Most beach seines use multifilament and in some cases monofilament nylon, but other synthetic materials may be used. Discarded fragments of nylon webbing sink. The derelict net fragments likely have low ghost fishing risk unless it ends up entangled on a 3-dimensional feature and other environmental conditions described under set and fixed gillnet exist. (0.2)<br><br>2. Discarded fragments of nylon netting can retain some ghost fishing efficiency for several years under certain conditions. However, the risk is relatively low unless discarded net fragments end up on 3-dimensional structures. (0.5)	Prado, 1990; FAO, 2001; Samoilys et al., 2011; Tietze et al., 2011; Bountiful Seines, 2020

	3. Discarded net fragments from this non-selective fishing gear have a very low ghost fishing catch risk for target pelagic species, and may have a low catch risk for target demersal species. As with set and fixed gillnets, under certain conditions the discarded monofilament netting from beach seines might result in ghost fishing of marketable demersal species and vulnerable species, although the risk is relatively low because derelict beach seines are likely to only result from discarded fragments of gear. (0.2)	
Seine, boat	1. Derelict fragments of synthetic boat seine netting, which may be made of polypropylene, or the same polyethylene twines as used by demersal trawl vessels, floats. Some boat seine fisheries, including small-scale artisanal boat seine fisheries, may use nylon twines, which sink. Demersal boat seine fisheries (including Danish seines – also called anchor seining, and Scottish seines – also called fly dragging), which exist in several European countries, Australia, Indonesia, Japan, Russia, Korea, Philippines and Canada, are a type of encircling gear that are dragged a small distance at a slow speed relative to other mobile gears such as bottom trawls. Unlike demersal trawls, boat seine vessels fish at grounds that are flat and smooth (the long towing ropes, up to 2 km in length, snag easily on boulders). Unwanted gear components may be discarded, or sections of net may be lost when the gear snags on boulders or debris on the seabed. The loss of an entire net is very unlikely to occur. (0.5)<br><br>2. Floating fragments of boat seine netting likely maintain ghost fishing efficiency for several years. Net fragments that occur on the seabed likely have minimal fishing efficiency. (0.5)<br><br>3. Net fragments on the seabed pose a low risk of ghost fishing. Vulnerable species could entangle in floating net fragments and possibly lines. There is a low risk of ghost fishing mortality of target species. (0.4)	Prado, 1990; FAO, 2001; Galbraith et al., 2004; Samoilys et al., 2011; Suuronen et al., 2012; Deshpande et al., 2020; O'Neill and Noack, 2020
Trawl, bottom	1. The loss of an entire net is a rare event, which might occur if the net snags on a bottom feature, but often breakaways and/or weak links are incorporated in the gear to prevent the loss of the entire gear when snagging on the substrate occurs. The lower panels or fragments of the lower panels of the trawl are frequently lost when the trawl snags on a bottom feature (on rough seabed or debris). In some regions, discarding damaged trawl components, including net fragments, at sea may be a common practice. Net fragments that remain on the seabed would have minimal ghost fishing catch risk. Floating trawl net fragments have a high risk of entangling large marine organisms. Bottom trawl netting is typically made of polyethylene and in some cases polypropylene, and netting fragments of these materials would initially float if not entangled on the seabed. (0.75)<br><br>2. Floating fragments of synthetic bottom trawl netting likely maintain ghost fishing efficiency for several years. The polyethylene netting is thick and stiff, so that the meshes remain open and do not quickly collapse, retaining ghost fishing efficiency for a long period. Net fragments that occur on the seabed may quickly lose any ghost fishing efficiency if it becomes incorporated into the hard substrate or buried in sediment. (0.75)<br><br>3. Fur seals, marine turtles, sharks, rays, marine mammals and other vulnerable species entangle in floating trawl net fragments and possibly lines. There is a low risk of ghost fishing mortality of target species in bottom trawl net fragments. (0.5)	Fowler, 1987; Prado, 1990; Jones, 1995; Donohue et al., 2001; Wilcox et al., 2014; GGGI, 2017; Atlantic Avitaillement, 2020; Deshpande et al., 2020; King Net, 2020; Suuronen et al., 2020

Trawl, midwater otter	1. Pelagic trawl netting may be made of polyethylene and polypropylene (which float), polyamide (which sinks), or a mix of these materials, however polyamide may be the most common net material in midwater trawls. If a floating trawl net fragment does not collapse into a mass, then it may pose a risk of entanglement. Net fragments that occur on seabed have minimal catch risk. The loss of an entire net is very unlikely to occur, and sections of the gear are also not typically lost as it is very rare to snag on the seabed or debris, but can occur on rare occasions. (0.4)<br><br>2. Floating fragments of synthetic midwater trawl netting likely maintain some ghost fishing efficiency for several years, although the catching efficiency would be very low if the meshes and net collapses. The polyamide twine is thin and not stiff, causing the meshes to close and lost net sections to quickly collapse. Net fragments that occur on the seabed likely have minimal fishing efficiency. (0.25)<br><br>3. The use of small meshes, relative to bottom trawls, results in a lower risk of entangling large organisms, but may have some catch risk of smaller organisms, including some vulnerable species such as seabirds, however the predominant use of thin line for twine reduces the catching efficiency of derelict netting. The net fragments are unlikely to catch target species of small pelagic schooling species (e.g., anchovy, herring, mackerel, capelin). (0.25)	Prado, 1990; Donohue et al., 2001; GGGI, 2017; Atlantic Avitaillement, 2020; Deshpande et al., 2020; King Net, 2020; Suuronen et al., 2020
Troll	Score of handline was applied. Anchored FADs are used by some troll fisheries, but likely a small proportion globally.	de San and Pages, 1998; Desurmont and Chapman, 2000; Widodo et al., 2016

S3.2. Distribution and transfer of toxins and microplastic into marine food webs

Table S3 assesses gear-specific relative risks from the dispersal and transfer of toxins, including persistent organic pollutants, and microplastic into marine food webs, including both chemical pollutants that leach from plastics and that are adsorbed to the surface of plastics. From Table 1, the following three metrics were assessed to determine gear-specific relative risks:

(1) Proportion of the volume of derelict gear that is made of plastic;
(2) Exposure to forces (abrasion, reactions from exposure to UV radiation - photolysis, photo-oxidation, thermo-oxidation, biodegradation) that cause plastic gear components to break down into microplastic (does the derelict gear float, sink in shallow areas or sink in deep areas); and
(3) Relative productivity of the habitat(s) where the ALDFG occurs (an indicator of the relative risk of incorporation of toxins and microplastic into food webs).

Table S3. Gear-specific estimates of the relative risk from the dispersal and transfer of toxins, including persistent organic pollutants, lead and microplastics, into marine food webs.

Gear	Rationale	Citations
Barrier, fence, weir, corral	1. Materials used for barriers, fences, weirs and corrals is variable. In some fisheries, the netting of the wings and bags nylon or other plastic material, as are floats and float lines if used. However, in some fisheries, natural materials such as plaited mats and palm fronds, are used. The frame and stakes or weights used to affix the trap to the seabed are typically not plastic. On average, we very roughly estimate that plastic makes up 70% of the volume of fyke net gear. (0.7)	FAO, 2001; Slack-Smith, 2001; Macfadyen et al., 2009; Samoilys et al., 2011; Fitri and

	2. Barriers, fences, weirs and corrals are typically installed in sheltered, shallow, coastal tidal habitats, including in coral reefs, seagrass beds, salt marshes and edges of mangroves of estuaries, lagoons and bays, where there is high UV and heat exposure, low mechanical stress from wave and current energy causing abrasion on the seabed, and relatively high microorganism and macrofauna local abundance. (1.0)<br><br>3. These traps can be installed in highly productive, shallow, coastal habitats. (1.0)	Pramonowibowo, 2015; Touhy et al., 2020
Fyke net	1. Materials used for fyke nets is variable. In some fisheries, the netting of the wings and bags are nylon, polyethylene – including Dyneema, or other plastic material, as are floats and float lines if used. However, in some fisheries, natural materials such as plaited mats and palm fronds, may be used. The frame and stakes or weights used to affix the trap to the seabed may be made of metal or wood. On average, we very roughly estimate that plastic makes up 70% of the volume of fyke net gear. (0.7)<br><br>2. Fyke nets are used in shallow coastal habitats, sometimes in areas with strong currents, such as at river and stream mouths in estuaries, as well as in shallow bays and other coastal habitats. On average, the fishing areas where the gear is deployed has some UV and heat exposure, high mechanical stress from wave and current energy causing abrasion on the seabed, and relatively high microorganism and macrofauna local abundance. (1.0)<br><br>3. These traps can be installed in highly productive, shallow, coastal habitats. (1.0)	FAO, 2001; Macfadyen et al., 2009; Samoilys et al., 2011; Oksanen et al., 2015; Mahi et al., 2018
Pot	1. A wide range of materials are used for the components of pots. Buoys and lines are typically plastic. Some designs use plastic frames, meshes (e.g., multifilament nylon, plastic coated steel rods, PVC covered wire) and plastic entrances, while others are made completely of natural materials (e.g., wooden frames with bamboo and rattan slats, ceramic). On average, we very roughly estimate that plastic makes up 30% of the volume of pot gear. (0.3)<br><br>2. Pots are deployed at a wide range of depths and habitat types, from shallow coastal habitats in estuaries and lagoons, to deep marine waters, some on the seabed, some midwater. Derelict pots that occur in shallow, high energy habitats have high exposure to forces that cause plastic gear components to break down into microplastic. (0.5)<br><br>3. ALDFG from pots that were set in shallow, coastal areas can occur in highly productive habitats, including coral reefs, seagrass beds and salt marshes, while ALDFG from pots that were set in deep water are more likely to affect less productive ecosystems. (0.5)	Stevens et al., 2000; FAO, 2001; Slack-Smith, 2001; Chiappone et al., 2002; Al-Masroori et al., 2004; Macfadyen et al., 2009; Samoilys et al., 2011; Uhrin and Schellinger 2011; Ahmadi et al., 2014; Galbraith et al., 2014; Uhrin et al., 2014; Prajith and Remesan, 2019; Ningtiyas et al., 2020; Stevens, 2020
Pound net	1. Netting may be made of nylon twine. In some fisheries, netting of pound nets could be made of natural materials. The frame and stakes are not plastic. On average, we very roughly estimate that plastic makes up 70% of the volume of pound net gear. (0.7)<br><br>2. Installed in shallow, coastal waters of up to about 20 m depth, including in lagoons, estuaries and bays, where there is high UV and heat exposure, low mechanical stress from wave and current energy causing abrasion on the seabed, and relatively high microorganism and macrofauna local abundance. (1.0)	Cheng and Chen, 1997; FAO, 2001; Abe and Shiode, 2009; Gilman, 2009; Macfadyen et al., 2009; Gilman et al., 2010; Silva et al., 2011

	3. Pound nets can be installed in highly productive, shallow, coastal habitats. (1.0)	
Gillnet, drift	1. Roughly estimated that 95% of the volume of a fleet (string) of gillnet panels (sheets) are typically made of plastic. The most common material for webbing in most gillnet fisheries is now monofilament. Buoys, floats, headropes, leadlines, buoy lines, skirtlines and bridles may also all be made of plastic materials). (0.95)<br><br>2. Derelict driftnets float and drift, with high UV and heat exposure and high mechanical stress from wave and wind energy and relatively high microorganism and macrofauna local abundance. (1.0).<br><br>3. Derelict gillnet webbing occurs in productive coastal habitats. Gillnets are used predominantly within 20 nm of shore. (1.0)	Mio et al., 1990; Donohue et al., 2001; He, 2006; CMS, 2011; Wilcox et al., 2014; Apriliani et al., 2019; Gyi, 2020; Stelfox et al., 2020
Gillnet, set and fixed; trammel net; combination gillnet/trammel net	1. As with driftnets, we roughly estimate that 95% of the volume of a fleet of panels of set and fixed gillnets and trammel nets are typically made of plastic, where the webbing is typically made of monofilament or multi-monofilament polyamide (nylon), and buoys, floats and lines are also made of plastic. (0.95)<br><br>2. The gear is deployed at a variety of depths and environments, but gillnets are predominantly used within 20 nm of shore. We very roughly estimate that 95% of the gear fishes in shallow coastal areas, where derelict gear would have relatively high UV exposure, high mechanical stress from wave and current energy causing abrasion on the seabed, and relatively high microorganism and macrofauna local abundance. (0.95)<br><br>3. We roughly estimate that 95% of derelict gear from set and fixed gillnets and trammel nets occurs in productive coastal habitats, and 10% occurs in relatively unproductive deep water habitats. (0.95)	MacMullen et al., 2003; CMS, 2011; Galbraith et al., 2014; Gilman et al., 2016; Deshpande et al., 2020; Gyi, 2020; King Net, 2020
Hand dredge, harpoon, spear, lance, tongs, rakes, hand-collected (including diving) - shore- and boat-based	1. Most of these coastal gears are made of metal or wood. Spearguns may have some plastic components, and harpoons and spears may have retrievable lines made of plastic, but these are a low risk of becoming ALDFG (e.g., see Frisch et al., 2008). (0.0)<br><br>2. Not applicable – no plastic components to be subject to forces that cause breakdown into microplastic. (0.0)<br><br>3. These gears are used in shallow, coastal areas, in relatively productive habitats. No plastic gear components are at risk of becoming derelict. Toxic metals (e.g., lead, zinc, cadmium) may be used. For example, spearfishers fishing at night might discard spent flashlight batteries, which may contain cadmium, lead, mercury, nickel and lithium. (0.05)	Barnette, 2001; FAO, 2001; Bjordal, 2002; Frisch et al., 2008; Karnchanawong and Limpiteeprakan, 2009; James et al., 2018; ODFW, 2019; JBL, 2020; Tuna Fish Tackle, 2020
Handline, midwater, surface and bottom	1. For most handline fisheries, plastic makes up >90% of the volume of ALDFG, including monofilament and braided polyamide line (mainline, branchlines, snoods), artificial lures if used, and floats, marker buoys and lines, if used. (0.9)<br><br>2. Fishing grounds are broad, including open ocean pelagic for tuna handlining, to deep water seamounts, to shallow, coastal areas. Derelict gear that sinks below the photic zone, in deep water habitat have relatively low exposure to forces that cause plastic gear components to break down into microplastic, while	Bjarnason, 1992; FAO, 2003; Zabka et al. 2006; AFMA, 2019a,b

	derelict gear that sinks in coastal, low energy environments would have relatively high UV and heat exposure, low mechanical stress from wave and current energy, and high microorganism and macrofauna local abundance. If floats and marker buoys are used, if derelict, these would have high exposure to forces that break down plastic into microplastic. (0.4)<br><br>3. ALDFG from handline fisheries occurring at open ocean fishing grounds would sink to relatively unproductive, deep water habitats, while derelict gear from fisheries occurring in shallow, coastal areas would be relatively productive. (0.4)	
Longline, demersal	1. The volume of complete demersal longline gear is ~90% plastic. Floats and floatlines are plastic, snoods (gangions) may be polyester or polyamide, including monofilament or braided nylon and twister polyester, the groundline, and mainline in double-line systems, may be nylon monofilament or polyester. (0.9)<br><br>2. Abandoned and lost demersal longlines are set and remain on or near the seabed. Discarded sections of mainline and discarded snoods, which are made of polyester and polyamide (nylon), sink. Derelict gear occurring in deep water, below the photic zone, would have no UV or heat exposure, low mechanical stress, low microorganism and macrofauna local abundance. Gear markers (e.g., floats, light buoy, high flyer, radio beacon), which make up a small component of ALDFG from demersal longline fisheries, would float and drift and have relatively high UV and heat exposure, mechanical stress from wave and wind energy, and relatively high microorganism and macrofauna local abundance. (0.1)<br><br>3. Abandoned and lost demersal longlines are set and would remain on the seabed in relatively unproductive habitat. Discarded sections of mainline and discarded snoods, which are made of polyester and nylon, sink in relatively unproductive deep water habitat. Derelict gear markers and lines would float and drift and might ground on sensitive coastal habitats. (0.1)	Natural Resource Consultants, 1990; Webber and Parker, 2012; Deshpande et al., 2020; Mustad Autoline, 2020
Longline, pelagic	1. The volume of complete pelagic longline gear is ~90% plastic. The plastic gear components include: monofilament polyamide mainline and branchlines, plastic floats, plastic floatlines, plastic chafing gear, in some cases lightsticks, flagpoles, radio buoys, line to connect radio buoys to the mainline, etc., and non-plastic components include: hooks, snaps, crimps, weighted and unweighted swivels, in some cases wire trace, etc. Discarded damaged line and bite-offs of terminal tackle may be >90% plastic by volume. (0.9)<br><br>2. Derelict gear markers (e.g., floats, buoys, radio buoys) from both industrial and artisanal fisheries, and lost and abandoned pelagic longlines, which is mainly a risk in small-scale artisanal fisheries that do not use radio buoys and other electronic technology to track the location of the gear, and by illegal vessels, would continue to float and drift, where there would be relatively high UV and heat exposure, mechanical stress from wave and wind energy, and relatively high microorganism and macrofauna local abundance. Derelict gear occurring in deep water would have no UV or heat exposure, low mechanical stress, low microorganism and macrofauna local abundance. (0.5)<br><br>3. Lost artisanal longlines could ground on sensitive coastal and nearshore habitats. Derelict pieces of monofilament line and terminal tackle would sink to deep water habitat. (0.5)	Beverly et al., 2003; Pacific Ocean Producers, 2019

Pole-and-line, including anchored FADs	1. Derelict sections of pole-and-line monofilament nylon line and hooks (excluding poles, which do not likely become derelict) are ~90% plastic by volume. Derelict anchored FADs have a wide variety of designs and materials, and on average may be about 30% plastic by volume. A large proportion of the catch from pole-and-line fisheries comes from fisheries that use anchored FADs. The number of FADs per pole-and-line vessel may be less than the ratio for tuna purse seine vessels (see Table S2). (0.35)<br><br>2. Monofilament line and hooks sink, where it is likely incorporated into the seabed. Surface and subsurface components and attached lines of derelict anchored FADs may float and drift, and eventually components of derelict anchored FADs sink, ground on coastlines, or could end up in floating garbage patches. Thus, the monofilament nylon lines have minimal exposure, and we estimate that about a third of the plastic components of derelict anchored FADs occur at the sea surface or ground on coastal habitats, where they have high exposure to forces that break macroplastics into microplastics of high UV and heat exposure, high mechanical stress from wave and wind energy and relatively high microorganism and macrofauna local abundance. (0.17)<br><br>3. Pole-and-line fishing for skipjack and albacore tunas is conducted primarily in offshore pelagic areas – derelict monofilament line and hooks sink primarily to deep, relatively unproductive habitat. Some pole-and-line fishing effort occurs at relatively productive shallow seamounts, where derelict gear may settle. Surface and subsurface components and attached lines of derelict anchored FADs may float and drift, and could sink and ground in productive habitats. (0.17)	de San and Pages, 1998; Desurmont and Chapman, 2000; Sibisopere, 2000; FAO, 2001; Samoilys et al., 2011; Beverly et al., 2012; Miller et al., 2016, 2017; Adam et al., 2019; ISSF and IPNLF, 2019; Proctor et al., 2019
Purse seine, non-tuna	1. Nets are typically made of polyamide (nylon) and polyester, and derelict fragments of netting are 100% plastic. (1.0)<br><br>2. Net fragments of polyamide and polyester sink in deep water, resulting in relatively low exposure to forces that cause plastic components to break down into microplastics, with no UV or heat exposure, low mechanical stress, low microorganism and macrofauna local abundance. (0.05)<br><br>3. Derelict purse seine net fragments likely end up in relatively unproductive, deep water habitats. (0.05)	Galbraith et al., 2014; Tang et al., 2018; Zhou et al., 2019; Deshpande et al., 2020; King Net, 2020; Netmark, 2020
Purse seine, tuna, including drifting and anchored FADs	1. About half of the catch by tuna purse seine fisheries comes from sets on drifting FADs. While some drifting FADs are made of biodegradable materials, such as bamboo, most are made of plastic, including nylon netting, as well as metal. Most drifting FADs are constructed of a mix of synthetic and biodegradable materials. Gilman et al. (2018) reported, "For example, the surface structure can include bamboo and other biodegradable materials, while components used to augment floatation are made of synthetic material. The appendage can include rope made of cotton but meshes made of synthetic compounds." And the attached satellite buoys and other instrumentation (echo sounders) are made of synthetic materials. A few tuna purse seine companies are participating in pilots and research of biodegradable drifting FADs. Tuna purse seine webbing is typically made of polyamide and polyester, i.e. 100% plastic, but fragments of the purse seine nets likely make up a small proportion of the derelict gear produced by this fishing method, as the majority of the volume of derelict gear is likely lost and abandoned FADs. (0.5)<br><br>2. Tuna purse seine webbing is typically made of polyamide and polyester, and fragments of this netting sink (however, see Stelfox et al. [2020] who reports observing drifting derelict net fragments from tuna	Maufroy et al., 2015; Escalle et al., 2017; ICCAT. 2017; Gilman et al., 2018; ISSF, 2019; Banks and Zaharia, 2020; Consoli et al., 2020; Sinopoli et al., 2020

	purse seine fisheries). About 66% of derelict drifting FADs, and possibly the same for anchored FADs, sink, about 5% to 10% ground on tropical coastal habitats, and presumably the remainder floats for a prolonged period. Thus, about a third of derelict gear from tuna purse seine fisheries occurs at or near the sea surface where it is exposed to forces that cause plastic components to break down into microplastic of high UV and heat exposure, high mechanical stress from wave and wind energy and relatively high microorganism and macrofauna local abundance. (0.3)<br><br>3. Deepwater habitats where derelict purse seine net fragments likely end up have relatively low productivity. Derelict polyethylene (nylon) ropes from anchored FADs used by purse seine vessels targeting mahimahi in the Mediterranean occurred in black coral (*Leiopathes glaberrima*) colonies. Derelict FADs constitute the majority of derelict gear from this fishing method. Discussed in Table S5, between 5% and 10% of deployed drifting fish aggregating devices (FADs) run aground on coastal habitats in the tropics, including on coral reefs. An estimated 66% of deployed drifting FADs sink. (0.3)	
Seine, beach	1. Seine nets are likely > 90% plastic by volume. The webbing is typically made of nylon, lines are made of nylon or polyethylene, leadline weights are various materials including lead, and poles are various materials including wood and PVC. (0.9)<br><br>2. Derelict net fragments and nylon lines likely sink in shallow coastal habitats (lagoon, bay, seagrass bed, mangrove wetland, estuary) where eventually, in low energy systems, they may be buried or become incorporated into the seabed. Derelict floats and polyethylene lines would initially float and drift, but are a low risk of becoming ALDFG. (0.5)<br><br>3. Derelict gear from beach seines occur in highly productive coastal and nearshore ecosystems. (1.0)	Prado, 1990; Samoilys et al., 2011; Tietze et al., 2011; Bountiful Seines, 2020
Seine, boat	1. ALDFG from boat seine gear is about 80% plastic by volume. (0.8)<br><br>2. Floating derelict seine netting may drift, and eventually might sink, ground on coastlines, or could end up in floating garbage patches. Perhaps half of the plastic in derelict boat seines float at the sea surface or ground on coastal habitats where they have relatively high exposure to forces that break macroplastics into microplastics of high UV and heat exposure, high mechanical stress from wave and wind energy and relatively high microorganism and macrofauna local abundance. (0.5)<br><br>3. Most boat seine fisheries occur from shallow waters less than 35 m to around 500 m in marine waters, targeting demersal fishes and deepwater shrimps, fishing at grounds with a flat and smooth seabed. A proportion of derelict floating boat seine nets could ground in productive coastal and nearshore habitats. (0.125)	Prado, 1990; FAO, 2001; Galbraith et al., 2004; Samoilys et al., 2011; Suuronen et al., 2012; Deshpande et al., 2020
Trawl, bottom	1. About 80% of the volume of ALDFG from bottom trawls is made of plastic. (0.8)<br><br>2. Bottom trawl netting, which is typically made of polyethylene and in some cases polypropylene, if not on the seabed, would initially float and have high exposure to forces that break the gear down into microplastic. Derelict gear is likely mainly lost lower panels or fragments of the lower panels of the trawl, and discarded damaged trawl components, including net fragments. If on the seabed there would be low exposure to forces that cause plastic to break down into microplastic. (0.75)	Jones, 1995; Sala et al., 2013; Galbraith et al., 2014; Lenoir et al., 2019; Atlantic Avitaillement, 2020; Deshpande et al., 2020; King Net, 2020; Suuronen et al., 2020

	3. Floating derelict bottom trawl net fragments could ground in productive coastal and nearshore habitats. Lost net fragments on the seabed, especially in deep water, would have low productivity. (0.75)	
Trawl, midwater otter	1. About 80% of the volume of ALDFG from midwater trawls is made of plastic. (0.8)<br><br>2. Polyamide midwater trawl net fragments, which sink, and polyethylene and polypropylene net fragments that over time become negatively buoyant and sink to the seabed, have minimal exposure. Polyethylene and polypropylene net fragments, which initially float when abandoned, lost or discarded, have high exposure to forces that cause plastic to break down into microplastic. Polyamide may be the predominant net material in midwater trawls. (0.4)<br><br>3. Fragments of netting on the seabed in deep water would have minimal adverse effects on habitat. A proportion of derelict pelagic trawl net fragments, when made of polyethylene, polypropylene or other materials that float, could ground in productive coastal and nearshore habitats. (0.4) s	Jones, 1995; Sala et al., 2013; Galbraith et al., 2014; Okuda and Nishida, 2017; Lenoir et al., 2019; Atlantic Avitaillement, 2020; Deshpande et al., 2020; King Net, 2020; Suuronen et al., 2020
Troll	1. Plastic makes up >90% of the volume of ALDFG from troll gear, on average, including monofilament polyamide and fluorocarbon (polyvinylidene difluoride) lines (both of which sink) and artificial lures/jigs if used. (0.9)<br><br>2. Fishing grounds are broad, including open ocean trolling for pelagics (e.g., tunas, mackerels), to shallow, nearshore areas to target coastal pelagic species (e.g., barracudas). Derelict gear that sinks in deep water habitat have relatively low exposure to forces that cause plastic gear components to break down into microplastic, while derelict gear that sinks in coastal, low energy environments would have relatively high UV and heat exposure, low mechanical stress from wave and current energy, and high microorganism and macrofauna local abundance. (0.4)<br><br>3. ALDFG from troll fisheries occurring at open ocean fishing grounds would sink to relatively unproductive, deep water habitats, while derelict gear from fisheries occurring in shallow, coastal areas would be relatively productive. (0.4)	Bjarnason, 1992; FAO, 2001, 2003; Seattle Marine and Fishing Supply, 2020

S3.3. Transporting invasive alien species and algae that cause red tides

Table S4 assesses gear-specific relative risks from the dispersal of invasive alien species (IAS) and microalgae that cause harmful algal blooms (HABs, red tides). From Table 1, the fishing gears were assessed to determine their relative risks based on whether the derelict gear initially floats or sinks.

Table S4. Gear-specific relative risk of ALDFG spreading invasive alien species and microalgae that cause HABs.

Gear	Rationale	Citations
Barrier, fence, weir, corral	These traps, when initially abandoned, would not float or change position. Storms would likely transport lost gear components over relatively small distances. Various materials are used for netting on barriers, fences, weirs and corrals. Some use webbing of polyamide and polyester, which sink. However, some use webbing of polyethylene and polypropylene, which float, and some use natural materials which can also	Prado, 1990; FAO, 2001; Slack-Smith, 2001; Macfadyen et al., 2009; Samoilys et al., 2011; Fitri and Pramonowibowo,

	float. Thus, discarded fragments of webbing from some traps might float. Derelict floating floats and float lines, if used, are another potential risk of transporting IAS and algae that cause red tides. (0.1)	2015; King Net, 2020; Touhy et al., 2020
Fyke net	Derelict fyke nets do not float. They may change position but only over relatively small distances. Float lines and buoys may become derelict and drift longer distances, if they are used. Various materials are used for netting on fyke nets. Some use webbing for the wings and bag made of polyamide and polyester, as well as wire and various other non-plastic materials, which sink. However, some use webbing of polyethylene – including Dyneema, and polypropylene, which float. Derelict floating floats and float lines, if used, are another potential risk of transporting IAS and algae that cause red tides. (0.1)	Prado, 1990; Oksanen et al., 2015; Mahi et al., 2018; King Net, 2020
Pot	As with fyke net traps, derelict pots do not float, but may change position over relatively small distances. Float lines and buoys may become derelict and drift longer distances. Various materials are used for meshes on pots. Some use webbing of polyamide and polyester, as well as wire and various other non-plastic materials, which sink. However, some use webbing of polyethylene and polypropylene, or natural materials (mangrove wood, bamboo, rattan) which may float. (0.1)	Prado, 1990; Slack-Smith, 2001; Al-Masroori et al., 2004; Samoilys et al., 2011; Ahmadi et al., 2014; Prajith and Remesan, 2019; King Net, 2020
Pound net	These traps, when initially abandoned, would remain where they were installed and not change position, or float. Storms would likely transport lost gear components over relatively small distances. Various materials are used for netting on pound nets. Some use webbing of polyamide and polyester, which sink. However, some use webbing of polyethylene and polypropylene, which float. (0.1)	Prado, 1990; Cheng and Chen, 1997; FAO, 2001; Abe and Shiode, 2009; Gilman, 2009; Gilman et al., 2010; Silva et al., 2011; King Net, 2020
Gillnet, drift	Abandoned and lost driftnets will initially float. Derelict buoys, floats and floatlines float. Discarded net fragments, now mainly made of monofilament, and most other individual components of driftnets, sink. (1.0)	Prado, 1990; He, 2006; Stelfox et al., 2020
Gillnet, set and fixed; trammel net; combination gillnet/trammel net	Monofilament and multi-monofilament polyamide are typically used for gillnet and trammel net webbing, and fragments of this netting sink. Floats, buoys and floatlines can become derelict and float, although this is a relatively low risk. If set (anchored) gillnets, which can be designed to fish anywhere from the surface, midwater to at or near the seabed, break from anchor lines, then the lost gear could float and drift, like a lost drift gillnet. (0.75)	Prado, 1990; FAO, 2001; MacMullen et al., 2003; Deshpande et al., 2020; King Net, 2020
Hand dredge, harpoon, spear, lance, tongs, rakes, hand-collected (including diving) - shore- and boat-based	These gears and components, if lost, abandoned or discarded, would sink. (0.0)	Barnette, 2001; FAO, 2001; Bjordal, 2002; Frisch et al., 2008; Karnchanawong and Limpiteeprakan, 2009; James et al., 2018; ODFW, 2019; JBL, 2020; Tuna Fish Tackle, 2020
Handline, midwater,	Derelict nylon lines and terminal tackle sinks. Derelict floats, marker buoys and lines, if used, would float. (0.02)	Prado, 1990; Bjarnason, 1992; FAO, 2001, 2003; AFMA, 2019a,b

surface and bottom		
Longline, demersal	Abandoned and lost demersal longlines are set and remain on or near the seabed. Discarded sections of mainline and discarded snoods, which are made of polyester and nylon, sink. Derelict gear markers (floats, light buoy, high flyer, radio beacon) and attached lines would float and drift. (0.1)	Prado, 1990; Webber and Parker, 2012; Deshpande et al., 2020; Mustad Autoline, 2020
Longline, pelagic	Lost and abandoned pelagic longlines would continue to float and drift, which is mainly a risk in small-scale artisanal fisheries that do not use radio buoys and other electronic technology to track the location of the gear. Discarded monofilament nylon branchlines, sections of mainline and terminal tackle sink. Derelict buoys, gear markers and attached lines would float and drift. (0.5)	Beverly et al., 2003; Pacific Ocean Producers, 2019
Pole-and-line, including anchored FADs	Surface and subsurface components and attached lines of derelict anchored FADs may float and drift. A large proportion of the catch from pole-and-line fisheries comes from fisheries that use anchored FADs; the number of FADs per pole-and-line vessel may be less than the ratio for tuna purse seine vessels (see Table S2). Derelict monofilament line and hooks from pole-and-line fisheries sink. (0.6)	de San and Pages, 1998; Desurmont and Chapman, 2000; Samoilys et al., 2011; Shainee and Leira, 2011; Beverly et al., 2012; Adam et al., 2019; ISSF and IPNLF, 2019; Proctor et al., 2019
Purse seine, non-tuna	Floats used on the purse seine net could become derelict, although likely a low risk. Non-tuna (anchovy, capelin, herring, sardine, etc.) purse seine nets are typically made of polyamide or polyester, and fragments of this netting sink. (0.1)	Prado, 1990; Galbraith et al., 2014; Tang et al., 2018; Zhou et al., 2019; Deshpande et al., 2020; King Net, 2020; Netmark, 2020
Purse seine, tuna, including drifting and anchored FADs	About half of the catch by tuna purse seine fisheries comes from sets on drifting FADs. Floats used on the purse seine net could become derelict, although likely a low risk. Tuna purse seine webbing is typically made of polyamide and polyester, and fragments of this netting sink (however, see Stelfox et al. [2020] who observed drifting derelict net fragments from tuna purse seine fisheries). Derelict drifting FADs float and drift. Rafts and anchor lines of derelict anchored FADs may float and drift. (0.9)	Prado, 1990; Itano, 2002; ICCAT, 2017; Gilman et al., 2018; Zhou et al., 2019; Atlantic Avitaillement, 2020
Seine, beach	Beach seines can be made of monofilament and multifilament nylon, and the netting sinks. Lines and ropes, which are made from nylon (sinks) or polyethylene (floats), and floats from the floatline could become derelict, but likely this is a low risk. (0.1)	Prado, 1990; Samoilys et al., 2011; Tietze et al., 2011
Seine, boat	Derelict fragments of synthetic boat seine netting, which may be made of polypropylene, or the same polyethylene twines as used by demersal trawl vessels, float. Some small-scale artisanal boat seines may use nylon twines, which sink. (0.5)	Prado, 1990; Galbraith et al., 2004; Samoilys et al., 2011; Suuronen et al., 2012; Deshpande et al., 2020
Trawl, bottom	Derelict gear from bottom trawls is likely mainly lost and abandoned net fragments, some floating at the surface, some on the seabed. Bottom trawl netting, which is typically made of polyethylene and in some cases polypropylene, if not on the seabed, would initially float. However, some demersal trawl nets are made of polyamide, which sinks. (0.75)	Prado, 1990; Sala et al., 2013; Galbraith et al., 2014; Lenoir et al., 2019; Atlantic Avitaillement,

		2020; Deshpande et al., 2020; King Net, 2020; Suuronen et al., 2020
Trawl, midwater otter	Pelagic trawl netting may be predominantly made of polyamide (which sinks), as well as polyethylene and polypropylene (which float), or a mix of these materials. (0.4)	Prado, 1990; Sala et al., 2013; Galbraith et al., 2014; Okuda and Nishida, 2017; Lenoir et al., 2019; Atlantic Avitaillement, 2020; Deshpande et al., 2020; King Net, 2020; Suuronen et al., 2020
Troll	Derelict nylon lines and terminal tackle sinks. (0.0)	Prado, 1990; Bjarnason, 1992; FAO, 2001, 2003; Seattle Marine and Fishing Supply, 2020

S3.4. Habitat Alteration and Degradation

Table S5 assesses gear-specific relative risks of habitat alteration and degradation by ALDFG. From Table 1, each gear category was assessed according to the relative risk of damaging sensitive habitats.

Table S5. Gear-specific relative risk of ALDFG altering and damaging habitat.

Gear	Rationale	Citations
Barrier, fence, weir, corral	Barriers, fences, weirs and corrals are typically installed in sheltered, shallow, coastal tidal habitats, including in coral reefs, seagrass beds, salt marshes and edges of mangroves of estuaries, lagoons and bays. Derelict fragments of netting, lines and weights from these traps could smother and abrade these sensitive coastal habitats. These traps can break away during large storms and cause substantial damage to coastal habitats, but are less likely to move position relative to fyke net and pot traps. (0.7)	FAO, 2001; Slack-Smith, 2001; Macfadyen et al., 2009; Samoilys et al., 2011
Fyke net	Fyke nets are used in shallow coastal habitats, sometimes in areas with strong currents, such as at river and stream mouths in estuaries, as well as in shallow bays and other coastal habitats. Derelict fyke nets can smother and abrade sensitive shallow, coastal habitats. Derelict fyke nets are more likely to be transported than more permanent traps, including pound nets, barriers, fences, weirs and corrals, but less likely to change position than pots. (0.9)	FAO, 2001; Macfadyen et al., 2009; Samoilys et al., 2011; Oksanen et al., 2015; Mahi et al., 2018
Pot	Pots are deployed at a wide range of depths and habitat types, from shallow coastal habitats in estuaries and lagoons, to deep marine waters, some on the seabed, some midwater. Derelict pots and buoy lines from gear that are set in shallow habitats can smother and abrade sensitive benthic habitats, including coral reefs, seagrass beds and salt marshes in estuaries and lagoons. ALDFG from pots that are set in deep-water habitat likely have minimal habitat impacts. (0.9)	Stevens et al., 2000; FAO, 2001; Slack-Smith, 2001; Chiappone et al., 2002; Al-Masroori et al., 2004; Macfadyen et al., 2009; Samoilys et al., 2011; Uhrin and Schellinger 2011; Ahmadi et al., 2014; Galbraith et

		al., 2014; Uhrin et al., 2014; Prajith and Remesan, 2019; Stevens, 2020
Pound net	Derelict pound nets could smother and abrade sensitive habitats. Pound nets are installed in shallow, coastal waters of up to about 20 m depth, including in lagoons, estuaries and bays, situated to intercept the migration pathways of target species. These traps can break away during large storms and cause substantial damage to coastal habitats, but are less likely to move position relative to fyke net and pot traps. (0.7)	Cheng and Chen, 1997; FAO, 2001; Abe and Shiode, 2009; Gilman, 2009; Macfadyen et al., 2009; Gilman et al., 2010; Silva et al., 2011
Gillnet, drift	Can smother and abrade coastal and benthic habitats. Monofilament webbing from gillnets is a predominant component of debris found in some tropical coral reefs and other coastal systems. (1.0)	Donohue et al., 2001; CMS, 2011; Wilcox et al., 2014; Gyi, 2020
Gillnet, set and fixed; trammel net; combination gillnet/trammel net	Can smother and abrade coastal and benthic habitats. Eventually becomes buried in soft bottom or incorporated into hard seabed, creating additional habitat. Monofilament webbing from gillnets is a predominant component of debris found in some tropical coral reefs and other coastal systems. (1.0)	Erzini et al., 1997; Macfadyen et al., 2009; CMS, 2011; Gilman et al., 2016; Gyi, 2020
Hand dredge, harpoon, spear, lance, tongs, rakes, hand-collected (including diving) - shore- and boat-based	ALDFG from these gears would likely sink to the seabed and pose limited risk of habitat damage. (0.0)	Barnette, 2001; FAO, 2001; Bjordal, 2002; Frisch et al., 2008; Karnchanawong and Limpiteeprakan, 2009; James et al., 2018; ODFW, 2019; JBL, 2020; Tuna Fish Tackle, 2020
Handline, midwater, surface and bottom	Derelict handline gear that is lost from snagging on bottom features likely has minimal risk of damaging habitat. Derelict monofilament and braided line could damage shallow, coastal sensitive habitats, and seamount habitat. Derelict floats, marker buoys and lines, if used, could ground on sensitive coastal habitats. Some handline fisheries fish at anchored FADs, but this may be the case for a small proportion of global handline fisheries. (0.3)	Bjarnason, 1992; FAO, 2001, 2003; Widodo et al., 2016; AFMA, 2019a,b
Longline, demersal	Lost and abandoned demersal longlines could damage benthic habitats, including demersal vulnerable marine ecosystems, including areas containing deep-water corals, sponge fields and seamounts. The mainline and snoods may become buried in soft bottom or become incorporated into hard seabed. Discarded gear components such as hooks, anchors, and sections of mainline and snoods, would sink and over time would likely pose a small risk to demersal habitats. Floats, marker buoys and lines, if lost or discarded, could drift and run aground on coastal habitats. (0.3)	Natural Resources Consultants, 1990; Fossa et al., 2002; Munoz et al., 2011; CCAMLR, 2012; Webber and Parker, 2012; Galbraith et al., 2014; Deshpande et al., 2020

Longline, pelagic	In-use gear may be lost in small scale artisanal fisheries not using radio buoys or other electronic technology to track the gear's position during the soak, and the gear could ground on vulnerable coastal habitats. When setting near shallow submerged features, gear can become lost by snagging on bottom features, including seamounts and reefs, documented for example on Cross Seamount near the main Hawaiian Islands. On very rare occasions, sections of mainline of active gear can be lost when entangled on large whales. Loss of terminal tackle due to bite-offs and severed and dropped branchlines, and discarded damaged gear components would sink likely in deep water and with little risk of damage to sensitive habitats. (0.4)	FAO, 2001; Gilman et al., 2012
Pole-and-line, including anchored FADs	Derelict monofilament nylon and hooks from pole-and-line fisheries, which sink primarily in deep waters but also at shallow seamounts, pose a relatively small risk of degrading habitat. Derelict lines and materials from the appendage and surface structure of anchored FADs used by pole-and-line vessels could run aground and damage sensitive coastal habitats. For example, anchored FADs used in Indonesian tuna fisheries use synthetic lines, where the line connecting the surface structure to cement blocks can be several km in length. A large proportion of the catch from pole-and-line fisheries comes from fisheries that use anchored FADs; the number of FADs per pole-and-line vessel may be less than the ratio for tuna purse seine vessels (see Table S2). (0.7)	de San and Pages, 1998; Desurmont and Chapman, 2000; Samoilys et al., 2011; Shainee and Leira, 2011; Beverly et al., 2012; Widodo et al., 2016; Miller et al., 2016, 2017; Adam et al., 2019; ISSF and IPNLF, 2019; Proctor et al., 2019
Purse seine, non-tuna	Purse seine net fragments of polyamide or polyester that sink to the seabed in deep water have a small risk of adversely affecting and altering benthic habitats. (0.05)	Galbraith et al., 2014; GGGI, 2017; Tang et al., 2018; Zhou et al., 2019; Deshpande et al., 2020; King Net, 2020; Netmark, 2020
Purse seine, tuna, including drifting and anchored FADs	About half of the catch by tuna purse seine fisheries comes from sets on drifting FADs. Between 5% and 10% of deployed drifting fish aggregating devices (FADs) run aground on coastal habitats in the tropics, including on coral reefs. An estimated 66% of deployed drifting FADs sink. Derelict polyethylene (nylon) ropes from anchored FADs used by purse seine vessels targeting mahimahi in the Mediterranean damaged black coral (*Leiopathes glaberrima*) colonies.<br>Tuna purse seine webbing is typically made of polyamide and polyester, and fragments of this netting sinks (however, see Stelfox et al. [2020] who observed drifting derelict net fragments from tuna purse seine fisheries). Purse seine net fragments that sink to the seabed in deep water likely cause minimal adverse effects on habitat. (0.9)	Maufroy et al., 2015; Escalle et al., 2017; GGGI, 2017; ICCAT, 2017; Gilman et al., 2018; ISSF, 2019; Banks and Zaharia, 2020; Consoli et al., 2020; Sinopoli et al., 2020
Seine, beach	If discarded in sensitive coastal and nearshore habitats, derelict beach seine nets and net fragments, which are made of nylon and sink, may cause habitat damage. (0.7)	Prado, 1990; Samoilys et al., 2011; Tietze et al., 2011; Bountiful Seines, 2020
Seine, boat	Floating net fragments could run aground on sensitive coastal habitats. Boat seines can occur in shallow waters less than 35 m to around 500 m, targeting demersal fishes and deep-water shrimps, fishing at grounds with a flat and smooth seabed. Some small-scale artisanal boat seines may use nylon twines,	FAO, 2001; Galbraith et al., 2004; Samoilys et al., 2011; Suuronen et al.,

	which sink, and likely pose minimal risk of habitat degradation, but may move around and damage demersal habitat. (0.5)	2012; Uzer et al., 2017; Deshpande et al., 2020
Trawl, bottom	Derelict gear from bottom trawls is likely mainly lost lower panels or fragments of the lower panels of the trawl and discarded damaged trawl components, including net fragments. Netting, which is typically made of polyethylene and in some cases polypropylene, if not entangled on the seabed, would initially float. Floating net fragments can run aground on sensitive coastal habitats. Trawl netting has been identified as a predominant component of debris found in some tropical coral reefs. However, some demersal trawl nets are made of polyamide (nylon), which sinks. Derelict trawl nets located on the seabed may move around and damage habitat. (0.75)	Donohue et al., 2001; Wilcox et al., 2014; GGGI, 2017; Deshpande et al., 2020
Trawl, midwater otter	Pelagic trawl netting may predominantly made of be made of polyamide (which sinks), but are also made of polyethylene and polypropylene (which float), or a mix of these materials. Floating net fragments could run aground on sensitive coastal habitats. Derelict midwater trawl netting that sinks to the seabed, due to having a smaller mesh size than bottom trawl nets, are likely to quickly accrete to the seabed, and be less likely to move around and damage benthic habitats, and lost midwater trawl nets are likely to occur in deep water with benthic habitats of relatively low vulnerability. Midwater trawl netting may be made of polyethylene or polyamide. (0.4)	Donohue et al., 2001; Wilcox et al., 2014; GGGI, 2017; Okuda and Nishida, 2017; Deshpande et al., 2020
Troll	If the derelict gear sinks in deep water, likely there is minimal risk of habitat damage. Derelict monofilament line could cause damage to shallow, coastal sensitive habitats. As with handline and pole-and-line fisheries, some troll fisheries fish at anchored FADs, but this may be the case for a small proportion of global troll fisheries. (0.25)	Bjarnason, 1992; FAO, 2001, 2003; Widodo et al., 2016

S3.5. Obstruction of In-use Fishing Gear, Navigation and Safety Risks at Sea

Table S6 assesses gear-specific relative risks of ALDFG obstructing in-use fishing gear, navigation and creating safety risks at sea. From Table 1, the following four metrics were assessed to determine gear-specific relative risks:

(1) Does the derelict gear initially float and have aerial overlap with marine vessels;
(2) Aerial overlap and encounterability with in-use fishing gear;
(3) Derelict gear materials; and
(4) Derelict gear visibility.

Table S6. Gear-specific relative risk of ALDFG obstructing in-use fishing gear, navigation and creating safety risks at sea.

Gear	Rationale	Citations
Barrier, fence, weir, corral	1. Abandoned barriers, fences, weirs and corrals would remain in place, affixed to the seabed in shallow waters, and would not float. Because the gear occupies the entire water column, from the seabed to the surface, these derelict traps would have the same risk of encountering and fouling marine vessels as in-use gear, which can be substantial if not adequately marked. Lost and discarded components of the traps would likely pose minimal risk of fouling vessels or in-use fishing gear. Derelict floating net fragments (if polyethylene and polypropylene are used for netting), and buoys and float lines if used, pose additional risk of fouling marine vessels. (0.25)	FAO, 2001; Slack-Smith, 2001; Macfadyen et al., 2009; Samoilys et al., 2011; Fitri and Pramonowibowo, 2015; Touhy et al., 2020

	2. Derelict barriers, fences, weirs and corrals, and components of the derelict gear, are unlikely to have aerial or vertical overlap with in-use gear. (0.0)<br><br>3. Floating derelict net fragments, and buoys and float lines if used, while unlikely to result from these traps, would pose a risk of fouling vessels and in-use surface fishing gear. (0.25)<br><br>4. Some derelict barriers, fences, weirs and corrals, if not adequately marked, could have low visibility and pose a risk of fouling vessels and in-use fishing gear, as is also the case for derelict floating net fragments, buoys and float lines if they occur. (0.5)	
Fyke net	1. Derelict fyke nets would be on the seabed in shallow water, with would have minimal risk of encountering and fouling marine vessels. Derelict floating buoys and float lines, if used, and derelict floating net fragments made of polyethylene, could occur in areas with marine vessels. (0.5)<br><br>2. There is a low risk of ALDFG from fyke nets having aerial or vertical overlap with in-use fishing gear. (0.0)<br><br>3. Floating derelict net fragments, and buoys and float lines if used, could pose some risk of fouling vessels and in-use surface fishing gear. (0.25)<br><br>4. Abandoned and lost fyke nets, affixed to the seabed in shallow water, may have low visibility but pose minimal risk of fouling marine vessels or in-use fishing gear. Derelict net fragments, buoys and line, which may float, would have low visibility, but assumed to be a low risk of being produced by fyke nets. (0.25)	FAO, 2001; Macfadyen et al., 2009; Samoilys et al., 2011; Oksanen et al., 2015; Mahi et al., 2018
Pot	1. Derelict floating buoys and float lines from pots could occur in areas with marine vessels and pose a risk of fouling marine vessels. (0.5)<br><br>2. Derelict pots could encounter in-use mobile fishing gears. Derelict floats and float lines pose a small risk of fouling surface fishing gears. (0.25)<br><br>3. Derelict floating buoys and float lines from pots would pose some risk of fouling vessels and in-use surface fishing gear (0.25)<br><br>4. Abandoned and lost pots would occur at the seabed and pose minimal risk of fouling marine vessels but might foul in-use mobile fishing gears. Derelict floating buoys and float lines may have relatively limited visibility during the day, and no visibility at night if not adequately marked. (0.375)	Stevens et al., 2000; FAO, 2001; Slack-Smith, 2001; Chiappone et al., 2002; Al-Masroori et al., 2004; Macfadyen et al., 2009; Samoilys et al., 2011; Uhrin and Schellinger 2011; Ahmadi et al., 2014; Galbraith et al., 2014; Uhrin et al., 2014; Prajith and Remesan, 2019; Stevens, 2020
Pound net	1. Abandoned pound nets would remain in place affixed to the seabed in shallow waters and would not float. Because the gear occupies the entire water column, from the seabed to the surface, these derelict traps would have the same risk of encountering and fouling marine vessels as in-use gear, which can be substantial if not adequately marked. Lost and discarded components of pound nets would likely sink and would likely pose minimal risk of fouling vessels or in-use fishing gear. (0.25)<br><br>2. There is a low risk of derelict pound nets having aerial or vertical overlap with in-use fishing gear. (0.0)	Cheng and Chen, 1997; FAO, 2001; Abe and Shiode, 2009; Gilman, 2009; Macfadyen et al., 2009; Gilman et al., 2010; Silva et al., 2011; Maryland DNR, 2020

	3. Fragments of netting from the leader, heart and pound that are made of nylon, which sinks, pose minimal risk of fouling marine vessels and in-use fishing gear. (0.0)<br><br>4. Abandoned pound nets would remain in place affixed to the seabed in shallow waters and would have the same risk of encountering and fouling marine vessels as in-use gear, which in some cases could be substantial if not adequately marked. Lost and discarded pound nets and components, which may sink, would have low visibility but pose minimal risk of fouling vessels or in-use fishing gear. (0.5)	
Gillnet, drift	1. Abandoned and lost driftnets will initially float. Derelict buoys, floats and floatlines float. Discarded net fragments, now mainly made of monofilament, and most other individual components of driftnets, sink. (1.0)<br><br>2. In-use fishing gear may encounter derelict floating driftnets, buoys and floatlines. Discarded net fragments pose minimal risk of encountering or fouling in-use fishing gear. (1.0)<br><br>3. Floatlines and floating masses of netting, mainly made of monofilament, pose a high risk of obstructing vessels by entanglement in propellers and shafts, and fouling in-use fishing gears. The risk of vessel damage from collisions with the derelict gear is low. (0.8)<br><br>4. Floating masses of driftnet netting, floats and floatlines would have low visibility, especially at night if not adequately marked. (1.0)	Eisenbud, 1985; Donohue et al., 2001; He, 2006; Wilcox et al., 2014; Stelfox et al., 2020
Gillnet, set and fixed; trammel net; combination gillnet/trammel net	1. Derelict floats and floatlines may encounter and foul marine vessels. Lost and abandoned set and fixed gillnets and trammel nets may be affixed to the seabed, where the floats and floatlines could encounter vessels. If set (anchored) gillnets, which can be designed to fish anywhere from the surface, midwater to at or near the seabed, break from anchor lines, then they might float and drift, like lost drift gillnets. Monofilament and multi-monofilament polyamide are typically used for gillnet and trammel net webbing, and discarded fragments of this netting sink. (0.8)<br><br>2. Mobile, bottom fishing gears may encounter lost and abandoned set and fixed gillnets and trammel nets on the seabed, and derelict set gillnets that are midwater or at the surface may encounter in-use fishing gears at these depths. Discarded net fragments and other components pose minimal risk of encountering or fouling in-use fishing gear. (0.8)<br><br>3. Derelict floating/drifting set gillnets, and derelict floats and floatlines pose a risk of fouling vessels by entanglement in propellers and shafts, and lost and abandoned gear might foul in-use, mobile, bottom fishing gears. There is minimal risk to vessels from collisions with the derelict gear. (0.8)<br><br>4. Floats, floatlines and marker buoys, and derelict floating/drifting set gillnets may have low visibility, in particular at night. Lost and abandoned nets, if not adequately marked, or if floatlines break or are cut, would not be visible to vessels using mobile, bottom, fishing gears. (0.8)	MacMullen et al., 2003; Gilman et al., 2016; Deshpande et al., 2020; King Net, 2020
Hand dredge, harpoon, spear, lance,	1. ALDFG from these gears would likely sink. (0.0)	Barnette, 2001; FAO, 2001; Bjordal, 2002; Frisch et al., 2008;

tongs, rakes, hand-collected (including diving) - shore- and boat-based	2. ALDFG from these gears likely sink in areas with very limited risk of encountering in-use fishing gear. (0.0)<br><br>3 and 4. Not applicable – the derelict gear is unlikely to encounter and poses minimal risk of fouling marine vessels or in-use fishing gear. (0.0)	Karnchanawong and Limpiteeprakan, 2009; James et al., 2018; ODFW, 2019; JBL, 2020; Tuna Fish Tackle, 2020
Handline, midwater, surface and bottom	1. Derelict handline gear (monofilament line and terminal tackle) sinks, posing no risk of encountering vessels. Derelict floats, marker buoys and lines, if used, could encounter marine vessels. (0.05)<br><br>2. Likely derelict handline gear on the seabed poses limited risk of encountering in-use fishing gear. Derelict floats, marker buoys and lines, if used, could encounter in-use surface fishing gears. (0.05)<br><br>3. Derelict gear of sections of monofilament polyamide line and terminal tackle, which would sink to the seabed, pose limited risk of fouling in-use fishing gear and no risk of fouling vessels. Derelict floats, marker buoys and lines, if used, could risk fouling in-use fishing gear and vessels. (0.05)<br><br>4. Derelict floats, marker buoys and lines may have relatively limited visibility during the day, and no visibility at night if not adequately marked, such as with radar reflectors. Derelict gear occurring at the seabed would not be visible, but would be unlikely to encounter marine vessels or in-use fishing gear. (0.05)	Bjarnason, 1992; FAO, 2001, 2003; AFMA, 2019a,b
Longline, demersal	1. Abandoned and lost demersal longlines are set and remain on or near the seabed, and discarded sections of mainline and discarded snoods, which are made of polyester and nylon, sink, posing no risk of encountering to marine vessels. Derelict buoys, gear markers (floats, light buoy, high flyer, radio beacon) and attached lines could encounter marine vessels. (0.1)<br><br>2. Derelict demersal longline gear on the seabed poses minimal risk of encountering in-use fishing gear. Derelict buoys, gear markers and attached lines could encounter in-use surface fishing gears. (0.1)<br><br>3. Buoys, gear markets and attached lines pose some risk of fouling marine vessels and in-use fishing gear, but low risk from vessel collisions. (0.25)<br><br>4. Buoys, gear markers and attached lines might have low visibility, especially at night and if no radar reflectors are attached. Derelict gear on the seabed would not be visible but poses no risk of encountering vessels and nominal risk of encountering and fouling in-use fishing gear. (0.1)	CCAMLR, 2012; Webber and Parker, 2012; Deshpande et al., 2020; Mustad Autoline, 2020
Longline, pelagic	1. Lost pelagic longlines, a risk primarily in artisanal fisheries not using radio buoys or other electronic technology to track the gear location while fishing and drifting, could encounter vessels. Derelict buoys and gear markers and attached lines could encounter vessels. Derelict sections of line and terminal tackle would sink and not encounter vessels. (0.5)<br><br>2. Lost floating and drifting pelagic longlines and derelict buoys and gear markers and attached lines could encounter in-use fishing gear. Derelict gear that sinks to the seabed poses no risk of encountering in-use gear. (0.5)	Beverly et al., 2003; Pacific Ocean Producers, 2019

	3. Lost floating and drifting pelagic longlines and derelict buoys and gear markers and attached lines risk fouling marine vessels and in-use surface fishing gears, but likely a low risk from vessel collisions. (0.5)<br><br>4. Buoys, gear markets and attached lines might have low visibility, especially at night and if no radar reflectors are attached. Derelict line and terminal tackle on the seabed would not be visible but poses no risk of encountering vessels and nominal risk of encountering and fouling in-use fishing gear. (0.1)	
Pole-and-line, including anchored FADs	1. Derelict anchored FADs' surface structure, appendage and lines will float and drift. A large proportion of the catch from pole-and-line fisheries comes from fisheries that use anchored FADs; the number of FADs per pole-and-line vessel may be less than the ratio for tuna purse seine vessels (see Table S2). Derelict pole-and-line monofilament and hooks sink. Derelict anchored FADs can occur in areas with marine vessels. (0.6)<br><br>2. Derelict anchored FADs can occur in areas with in-use fishing gear. Derelict pole-and-line monofilament and hooks sink and have minimal risk of encountering and fouling in-use fishing gear. (0.6)<br><br>3. Some designs of anchored FADs can pose a risk of entanglement in propellers and colliding with small vessels, and can foul in-use surface fishing gear. (0.6)<br><br>4. Derelict anchored FADs are a risk for entanglement in propellers and shafts, and colliding with small vessels, especially at night, and especially if the FADs are not lit and do not have radar reflectors. (0.6)	de San and Pages, 1998; Desurmont and Chapman, 2000; Samoilys et al., 2011; Shainee and Leira, 2011; Beverly et al., 2012; Widodo et al., 2016; Miller et al., 2016, 2017; Adam et al., 2019; ISSF and IPNLF, 2019; Proctor et al., 2019
Purse seine, non-tuna	1. Net fragments of polyamide and polyester sink. (0.0)<br><br>2. Likely net fragments sink in deep water and have no risk of encountering in-use fishing gear. (0.0)<br><br>3 and 4. Not applicable – the derelict gear is unlikely to encounter marine vessels or in-use fishing gear. (0.0)	Tang et al., 2018; Zhou et al., 2019; Deshpande et al., 2020; King Net, 2020; Netmark, 2020
Purse seine, tuna, including drifting and anchored FADs	1. Net fragments of polyamide and polyester sink (however, see Stelfox et al. [2020] who observed drifting derelict net fragments from tuna purse seine fisheries). About half of the catch by tuna purse seine fisheries comes from sets on drifting FADs. Derelict floating drifting and anchored FADs can occur in areas with marine vessel traffic. (0.8)<br><br>2. Likely net fragments sink in deep water and have no risk of encountering in-use fishing gear. Floating derelict FADs may overlap with fishing grounds and encounter surface fishing gears. (0.8)<br><br>3. Predominant designs of derelict drifting FADs, and many designs of anchored FADs that have broken their mooring, pose a risk of entanglement in propellers and colliding with small vessels, and can foul in-use surface fishing gear. (0.8)<br><br>4. Derelict net fragments likely sink. Derelict FADs with lights, radar reflectors, and day marks might be seen at day and night. Some drifting FADs are likely visible, but some are intentionally designed to have limited visual detection, with subsurface rafts and only floats at the surface. Derelict anchored FADs are a	Itano, 2002; Beverly et al., 2012; ICCAT. 2017; Gilman et al., 2018; Zhou et al., 2019; Atlantic Avitaillement, 2020

	risk for entanglement in propellers and colliding with small vessels, especially at night, and especially if the FADs are not lit and do not have radar reflectors. (0.7)	
Seine, beach	1. Beach seines can be made of monofilament and multifilament nylon, and thus fragments of netting sink. Lines and ropes, which are made from nylon (sinks) or polyethylene (floats), and floats from the floatline could become derelict, but likely a low risk. (0.1)<br><br>2. Derelict beach seine nets are unlikely to encounter in-use fishing gear. (0.0)<br><br>3 and 4. Not applicable – the derelict gear is unlikely to encounter marine vessels or in-use fishing gear. (0.0 for both)	Prado, 1990; Samoilys et al., 2011; Tietze et al., 2011
Seine, boat	1. Derelict fragments of synthetic boat seine netting, which may be made of polypropylene or the same polyethylene twines as used by demersal trawl vessels, float and can occur in areas that overlaps with marine vessel traffic. Some small-scale artisanal boat seines may use nylon twines, which sink. (0.5)<br><br>2. Floating seine netting may encounter in-use surface fishing gears. Nylon netting that sinks very likely would not encounter in-use fishing gear. (0.5)<br><br>3. Boat seine fragments of webbing are a high risk of fouling vessels and may risk fouling of in-use surface fishing gear. Seine nets made of nylon that sink pose a low risk of fouling vessels and in-use fishing gear. (0.5)<br><br>4. Floating boat seine floating net fragments are more difficult to detect that derelict gear with attached floats, but more visible than subsurface derelict gear. Derelict boast seine gear that sinks poses limited risk of fouling vessels and in-use gear. (0.5)	Prado, 1990; Galbraith et al., 2004; Samoilys et al., 2011; Suuronen et al., 2012; Deshpande et al., 2020; O'Neill and Noack, 2020
Trawl, bottom	1. Derelict gear from bottom trawls is likely mainly lost lower panels or fragments of the lower panels of the trawl and discarded damaged trawl components, including net fragments. Derelict fragments of bottom trawl netting, which is typically made of polyethylene and in some cases polypropylene, if not on the seabed, would initially float, and may overlap marine vessel traffic. (0.75)<br><br>2. Derelict net fragments on the seabed would not likely encounter in-use fishing gear. Floating trawl net fragments may encounter in-use surface fishing gears. (0.75)<br><br>3. Bottom trawl net fragments at the seabed are of minimal risk of fouling vessels and in-use fishing gear, but floating net fragments are a high risk. (0.75)<br><br>4. Floating trawl net fragments are more difficult to detect than derelict gear with attached floats, but more visible than subsurface derelict gear. Derelict gear on the seabed is not visible but no risk to vessels and minimal risk to in-use fishing gear. (0.75)	Prado, 1990; GGGI, 2017; Atlantic Avitaillement, 2020; Deshpande et al., 2020; King Net, 2020
Trawl, midwater otter	1. Pelagic trawl netting may predominantly made of be made of polyamide (which sinks), but are also made of polyethylene and polypropylene (which float), or a mix of these materials. If derelict trawl net fragments float, then they may overlap marine vessel traffic. (0.4)	Prado, 1990; GGGI, 2017; Okuda and Nishida, 2017; Atlantic Avitaillement, 2020;

	2. Floating trawl net fragments may overlap with fishing grounds and encounter in-use surface fishing gears. Derelict midwater trawl netting on the seabed would not likely encounter in-use fishing gear. (0.4)<br><br>3. Floating trawl net fragments, if large enough, could be a high risk of fouling vessels and may risk fouling of in-use surface fishing gear. Net fragments made of polyamide will sink and pose minimal risk of fouling vessels and in-use fishing gear. (0.4)<br><br>4. Net fragments that sink pose minimal risk of fouling vessels or fishing gear. Floating trawl net fragments are more difficult to detect that derelict gear with attached floats, but more visible than subsurface derelict gear. (0.4)	Deshpande et al., 2020; King Net, 2020
Troll	1. Derelict troll gear (monofilament line and terminal tackle) sinks, posing no risk of encountering vessels. (0.0)<br><br>2. Likely derelict troll gear sinks in areas with limited risk of encountering in-use fishing gear. (0.0)<br><br>3. Derelict gear is sections of monofilament polyamide line and terminal tackle, which would sink to the seabed, posing limited risk of fouling in-use fishing gear and no risk of fouling vessels. (0.0)<br><br>4. Occurring at the seabed, the derelict gear would not be visible, but the derelict gear is unlikely to encounter marine vessels or in-use fishing gear. (0.0)	Bjarnason, 1992; FAO, 2001, 2003

S3.6. Reduced Aesthetic and Use Value of Coastal and Nearshore Areas

Table S7 assesses gear-specific relative risks of ALDFG reducing the aesthetic and use value of coastal and nearshore areas. From Table 1, the following two metrics were assessed to determine gear-specific relative risks:

(1) Risk that the derelict gear will occur on coastlines and nearshore shallow habitats with socioeconomic value, and
(2) The proportion of the volume of the derelict gear that might occur on coastal and nearshore areas that is not made of natural and biodegradable materials

Table S7. Gear-specific relative risk of ALDFG reducing the aesthetic and use value of coastal habitats.

Gear	Rationale	Citations
Barrier, fence, weir, corral	1. Barriers, fences, weirs and corrals are typically installed in sheltered, shallow, coastal tidal habitats, including in coral reefs, seagrass beds, salt marshes and edges of mangroves of estuaries, lagoons and bays. Derelict barriers, fences, weirs and corrals and fragments of netting and other components of the gear would likely remain close to the site where the trap was installed, but fragments could be transported to coastlines and other nearshore areas valued for human uses. (0.25)<br><br>2. Materials used for these traps is variable. The netting is typically nylon or other plastic material, as are floats and float lines if used, while, in some fisheries, the frame and stakes or weights used to affix the trap to the seabed may be made of natural materials. We very roughly estimate that globally, 20% of the volume of barriers, fences, weirs and corrals is made of natural and biodegradable materials. (0.8)	FAO, 2001; Slack-Smith, 2001; Macfadyen et al., 2009; Samoilys et al., 2011

Fyke net	1. Fyke nets are used in shallow coastal habitats, sometimes in areas with strong currents, such as at river and stream mouths in estuaries, as well as in shallow bays and other coastal habitats. Derelict fyke nets and components of the gear, including net fragments, floats and float lines, could be transported to and ground on coastal and nearshore habitats. (0.5)<br><br>2. Materials used for fyke nets is variable. The netting of the wings and bags are typically nylon, polyethylene or other plastic material, as are floats and float lines if used, while the frame and stakes or weights used to affix the trap to the seabed are typically metal or wood. We very roughly estimate that globally, 20% of the volume of fyke nets is made of natural and biodegradable materials. (0.8)	FAO, 2001; Macfadyen et al., 2009; Samoilys et al., 2011; Oksanen et al., 2015; Mahi et al., 2018
Pot	1. Derelict pots and buoy lines can be transported and ground in various coastal and nearshore habitats, including coral reefs, seagrass beds and salt marshes, and can also occur on wrecks, including popular dive sites. (0.6)<br><br>2. Materials used for pots is highly variable. We very roughly estimate that globally, 20% of the volume of pots, lines and floats, is made of natural and biodegradable materials. (0.8)	Stevens et al., 2000; FAO, 2001; Slack-Smith, 2001; Chiappone et al., 2002; Al-Masroori et al., 2004; Macfadyen et al., 2009; Samoilys et al., 2011; Uhrin and Schellinger 2011; Ahmadi et al., 2014; Galbraith et al., 2014; Uhrin et al., 2014; Prajith and Remesan, 2019; Stevens, 2020
Pound net	1. Pound nets are installed in shallow, coastal waters of up to about 20 m depth, including in lagoons, estuaries and bays, situated to intercept the migration pathways of target species. Derelict pound nets and fragments of netting and other components of the gear would likely remain close to the site where the trap was installed, but fragments could be transported to coastlines and other nearshore areas valued for human uses. (0.25)<br><br>2. We very roughly estimate that globally, 10% of the volume of pound nets is made of natural and biodegradable materials. (0.9)	Cheng and Chen, 1997; FAO, 2001; Abe and Shiode, 2009; Gilman, 2009; Macfadyen et al., 2009; Gilman et al., 2010; Silva et al., 2011
Gillnet, drift	1. Derelict gillnet webbing occurs in coastal and nearshore habitats. (1.0)<br><br>2. From Table S3, ca. 95% of the volume of derelict gear is made of plastic and other non-biodegradable materials. (0.95)	Donohue et al., 2001; He, 2006; Wilcox et al., 2014; Stelfox et al., 2020
Gillnet, set and fixed; trammel net; combination gillnet/trammel net	1. Derelict gillnet webbing occurs in coastal and nearshore habitats. (1.0)<br><br>2. From Table S3, ca. 95% of the volume of derelict gear is made of plastic and other non-biodegradable materials. (0.95)	Erzini et al., 1997; MacMullen et al., 2003; Deshpande et al., 2020; King Net, 2020
Hand dredge, harpoon,	1. There is extremely minimal risk of ALDFG from these gears occurring on coastal and nearshore areas. (0.0)	Barnette, 2001; FAO, 2001; Bjordal, 2002;

spear, lance, tongs, rakes, hand-collected (including diving) - shore- and boat-based	2. Components of these gears are mainly made of metal and wood – we roughly estimate that 10% of these gears are synthetic. (0.1)	Frisch et al., 2008; Karnchanawong and Limpiteeprakan, 2009; James et al., 2018; ODFW, 2019; JBL, 2020; Tuna Fish Tackle, 2020
Handline, midwater, surface and bottom	1. Lost terminal tackle from snagging on bottom features would not likely adversely affect socioeconomic uses of coastal and nearshore areas. Derelict floats, marker buoys and lines, if used, could drift and ground on coastal habitats. ALDFG from coastal, nearshore handline fisheries could occur in areas important for other socioeconomic uses, but ALDFG from deep water, seamount and open ocean handline fisheries would sink far from coastal areas. (0.1)<br><br>2. For most handline fisheries, plastic makes up >90% of the volume of the gear. (0.9)	Bjarnason, 1992; FAO, 2001, 2003; AFMA, 2019a,b
Longline, demersal	1. Floats, marker buoys and attached lines, if lost or discarded, could drift and ground on or sink in coastal and nearshore habitats. These gear components are a small proportion of total ALDFG from this gear type. (0.05)<br><br>2. Floats, marker buoys and attached lines are mainly made of plastic materials. (1.0)	Webber and Parker, 2012; Deshpande et al., 2020; Mustad Autoline, 2020
Longline, pelagic	1. Lost floating pelagic longlines and derelict buoys and gear markers and attached lines could drift to and ground on or sink in coastal and nearshore habitats. (0.33)<br><br>2. Pelagic longline gear components are mostly made of plastic. (0.9)	Beverly et al., 2003; Pacific Ocean Producers, 2019
Pole-and-line, including anchored FADs	1. Surface and subsurface components and attached lines of derelict anchored FADs may ground on coastal habitats, including areas valued for human uses. A large proportion of the catch from pole-and-line fisheries comes from fisheries that use anchored FADs; the number of FADs per pole-and-line vessel may be less than the ratio for tuna purse seine vessels (see Table S2). Derelict monofilament nylon and hooks from pole-and-line fisheries sinks primarily in deep areas of the open ocean as well as over shallow seamounts, but not in areas used for human activities. (0.6)<br><br>2. See Table S3: Derelict sections of pole-and-line monofilament nylon line and hooks are roughly estimated to be >90% plastic by volume, but are not likely to occur on coastal or nearshore habitats. Derelict anchored FADs have a wide variety of designs and materials, and globally, on average, 30% of the volume of the anchored FAD is roughly estimated to be made of biodegradable materials. A large proportion of the catch from pole-and-line fisheries comes from fishing on anchored FADs (see Table S2). (0.7)	de San and Pages, 1998; Desurmont and Chapman, 2000; Samoilys et al., 2011; Shainee and Leira, 2011; Beverly et al., 2012Adam et al., 2019; ISSF and IPNLF, 2019; Proctor et al., 2019
Purse seine, non-tuna	1. Net fragments of polyamide and polyester likely sink in deep water with little risk of affecting nearshore and coastal habitats. (0.0)<br><br>2. Nets are typically made of polyamide (nylon) and polyester, and close to 100% of the gear is made of plastic (but almost no risk of the derelict gear reaching coastal areas). (1.0)	Tang et al., 2018; Zhou et al., 2019; Deshpande et al., 2020; King Net, 2020; Netmark, 2020
Purse seine, tuna, including	1. Derelict drifting and anchored FADs can litter coastlines, including areas valued for human uses. About half of the catch by tuna purse seine fisheries comes from sets on drifting FADs. (0.8)	Maufroy et al., 2015; Escalle et al., 2017;

drifting and anchored FADs	2. See Table S3. While some drifting FADs are made of biodegradable materials, such as bamboo, most are made of plastic, including nylon netting, as well as metal. Most drifting FADs are constructed of a mix of synthetic and biodegradable materials. Gilman et al. (2018) reported, “For example, the surface structure can include bamboo and other biodegradable materials, while components used to augment floatation are made of synthetic material. The appendage can include rope made of cotton but meshes made of synthetic compounds.” And the attached satellite buoys and other instrumentation (echo sounders) are made of synthetic materials. A few tuna purse seine companies are participating in pilots and research of biodegradable drifting FADs. Discussed above in pole-and-line gear, about 30% of the volume of anchored FADs is roughly estimated to be made of biodegradable materials. (0.7)	ICCAT, 2017; Gilman et al., 2018; ISSF, 2019; Banks and Zaharia, 2020
Seine, beach	1. Derelict fragments of beach seine nets would sink, would occur in shallow areas on the seabed of coastal and nearshore habitats where there would be some risk of reducing the socioeconomic value, including aesthetics and uses, of these areas. (0.5)<br><br>2. Other than the poles, beach seines are made of synthetic materials. (1.0)	Prado, 1990; Samoilys et al., 2011; Tietze et al., 2011; Bountiful Seines, 2020
Seine, boat	1. Floating boat seine net fragments could run aground on coastal habitats. Some small-scale artisanal boat seines may use nylon twines, which sink. (0.5)<br><br>2. Boat seine gear is made almost entirely of synthetic materials, and net fragments that would end up in coastal environments are entirely plastic. (1.0)	FAO, 2001; Galbraith et al., 2004; Suuronen et al., 2012; Uzer et al., 2017; Deshpande et al., 2020
Trawl, bottom	1. Derelict gear from bottom trawls is likely mainly lost lower panels or fragments of the lower panels of the trawl and discarded damaged trawl components, including net fragments. Bottom trawl netting, which is typically made of polyethylene and in some cases polypropylene, if not on the seabed, would initially float and could ground on coastlines. In some areas, trawl net fragments and ropes can be abundant on coastlines. (0.75)<br><br>2. Trawl net fragments are made of synthetic and non-biodegradable material. (1.0)	Jones, 1995; Donohue et al., 2001; Sala et al., 2013; Atlantic Avitaillement, 2020; Deshpande et al., 2020; King Net, 2020; Suuronen et al., 2020
Trawl, midwater otter	1. Pelagic trawl netting may predominantly made of be made of polyamide (which sinks), but are also made of polyethylene and polypropylene (which float), or a mix of these materials. In some areas, trawl net fragments and ropes have been observed to be abundant on coastlines. Midwater trawl netting may be made of polyethylene, and fragments of netting of this material will float and poses a risk of grounding on coastal habitats, but more likely are made of polyamide, and fragments of netting of this material will sink and are not a risk of occurring in coastal habitats. (0.4)<br><br>2. Trawl net fragments are made of synthetic and non-biodegradable material. (1.0)	Jones, 1995; Donohue et al., 2001; Sala et al., 2013; Okuda and Nishida, 2017; Atlantic Avitaillement, 2020; Deshpande et al., 2020; King Net, 2020; Suuronen et al., 2020
Troll	1. Lost terminal tackle that settles to the seabed would not likely adversely affect socioeconomic uses of coastal and nearshore areas. ALDFG from coastal, nearshore troll fisheries could occur in areas important for other socioeconomic uses, but ALDFG from offshore troll fisheries would sink far from coastal areas. (0.05)<br><br>2. Plastic makes up >90% of the volume of the gear. (0.9)	Bjarnason, 1992; FAO, 2001, 2003; Seattle Marine and Fishing Supply, 2020

S4. REFERENCES

Abe, O., Shiode, D. 2009. Development of sea turtle bycatch mitigation measures for Japanese pound net fisheries: A design concept to release turtles spontaneously. In: Gilman, E. (Ed.). *Proceedings of the Technical Workshop on Mitigating Sea Turtle Bycatch in Coastal Net and Trap Fisheries*. ISBN: 1-934061-40-9. IUCN, Western Pacific Regional Fishery Management Council, Southeast Asian Fisheries Development Center, Indian Ocean – South-East Asian Marine Turtle MoU, U.S. National Marine Fisheries Service, Southeast Fisheries Science Center, Gland, Switzerland; Honolulu, Bangkok, and Pascagoula, USA, pp. 43–44.

Adam, M., Jauhary, A., Azheem, M., Jaufer, A. 2019. *Use of Anchored FADs in the Maldives – Notes for a Case Study for Assessing ALDFG*. IOTC-2019-WPTT21-58. Indian Ocean Tuna Commission, Victoria Mahe, Seychelles.

AFMA. 2019a. *Droplines*. Available online, https://www.afma.gov.au/fisheries-management/methods-and-gear/droplines, accessed 5 Nov. 2020. Australian Fisheries Management Authority, Canberra.

AFMA. 2019b. *Power Handlines*. Available online, https://www.afma.gov.au/fisheries-management/methods-and-gear/power-handlines, accessed 5 Nov. 2020. Australian Fisheries Management Authority, Canberra.

Ahmadi, Rachman, M., Irhamsyah, Husin, S. 2014. Comparison of catching efficiency of two Indonesian traditional traps, Ayunan and Tamba. *Journal of Fisheries* 2: 113-118.

Al-Masroori, H., Al-Oufi, H., McIlwain, J., McLean, E. 2004. Catches of lost fish traps (ghost fishing) from fishing grounds near Muscat, Sultanate of Oman. *Fisheries Research* 69: 407-414.

Anderson RC, Waheed A. 1990. *Exploratory Fishing for Large Pelagic Species in the Maldives*. Available online, http://www.fao.org/3/AD835E/AD835E00.htm. Report BOBP/REP/46. Bay of Bengal Programme for Fisheries Development, Madras, India.

Antonelis, K., Huppert, D., Velasquez, D., June, J. 2011. Dungeness crab mortality due to lost traps and a cost-benefit analysis of trap removal in Washington State waters of the Salish Sea. *North Am. J. Fish. Manag*. 31: 880–893.

Apriliani, I., Hasan, Z., Junianto, Kusnadi, N. 2019. Characteristics of gillnet fishing gear and vessel construction in fish landing base of Karangsong, Indramayu, Indonesia. *World News of Natural Sciences* 23: 75-83.

Arthur, C., Sutton-Grier, A., Murphy, P., Bamford, H. 2014. Out of sight but not out of mind: Harmful effects of derelict traps in selected U.S. coastal waters. *Marine Pollution Bulletin* 86: 19-28.

Atlantic Avitaillement. 2020. *Webbing for Fishing Nets*. Available online, https://www.atlantic-avitaillement.com/webbing-for-fishing-nets, accessed 13 Sept 2020. Atlantic Avitaillement, AKX Group, Quimper, France.

Ayaz, A., Ünal, V., Acarli, D., Altinagac, U. 2010. Fishing Gear Losses in the Gökova Special Environmental Protection Area (SEPA), eastern Mediterranean, Turkey. *Journal of Applied Ichthyology* 26, 3.

Baeta, F., Jose Costa, M., Cabral, H. 2009. Trammel net's ghost fishing off the Portugueses central coast. *Fish. Res*. 98, 33–39.

Balderson, S., Martin, L. 2015. *Environmental Impacts and Causation of 'Beached' Drifting Fish Aggregating Devices around Seychelles Islands: A Preliminary Report on Data Collected by Island Conservation Society*. IOTC-2015-WPEB11-39. Indian Ocean Tuna Commission, Mahe, Seychelles.

Barnette, M. 2001. *A Review of the Fishing Gear Utilized within the Southeast Region and their Potential Impacts on Essential Fish Habitat*. Southeast Regional Office, National Marine Fisheries Service, St. Petersburg, Florida.

Banks, R., Zaharia, M. 2020. *Characterization of the Costs and Benefits Related to Lost and/or Abandoned Fish Aggregating Devices in the Western and Central Pacific Ocean*. Poseidon Aquatic Resources Management, Ltd., Hampshire, UK.

Beverly, S., Chapman, L., Sokimi, W. 2003. *Horizontal Longline Fishing Methods and Techniques. A Manual for Fishermen*. ISBN 982-203-937-9. Secretariat of the Pacific Community, Noumea, New Caledonia.

Beverly, S., Griffiths, D., Lee, R. 2012. *Anchored Fish Aggregating Devices for Artisanal Fisheries in South and Southeast Asia: Benefits and Risks*. Food and Agriculture Organization of the United Nations, Regional Office for Asia and the Pacific, Bangkok.

Bilkovic, D., Havens, K., Stanhope, D., Angstadt, K. 2014. Derelict fishing gear in Chesapeake Bay, Virginia: Spatial patterns and implications for marine fauna. *Marine Pollution Bulletin* 80: 114-123.

Bjarnason, B. 1992. *Handlining and Squid Jigging*. FAO Training Series No. 23. ISBN 92-5-103100-2. Food and Agriculture Organization of the United Nations, Rome.

Bjordal, A. 2002. The use of technical measures in responsible fisheries: Regulation of fishing gear. IN FAO. *A Fishery Manager's Guidebook*. FAO Fisheries Technical Paper 424. Food and Agriculture Organization of the United Nations, Rome, pp.21 -47.

Blasi, M., Roscioni, F., Mattei, D. 2016. Interaction of loggerhead turtles (Caretta caretta) with traditional fish aggregating devices (FADs) in the Mediterranean Sea. *Herpetological Conservation and Biology* 11: 386-401.

Bountiful Seines. 2020. *Beach Seines*. Available online, https://www.beachseines.com/, accessed 22 Sept. 2020. Bountiful Seines, Panama City, Florida.

Breen, P. 1989. A review of ghost fishing by traps and gillnets. In *Proceedings of the Second International Conference on Marine Debris,* 2, 571p.

Breen, P. 1990. A review of ghost fishing by traps and gillnets. In: Shomura, R., Godfrey, M. (Eds.). *Proceedings of the Second International Conference on Marine Debris, 2-7 April 1989, Honolulu, Hawaii*. US Department of Commerce, NOAA Tech Memo NMFS, NOAA-TM-NMFS-SWFSC-154, pp. 571-599.

Bullimore, B., Newman, P., Kaiser, M., Gilbert, S., Lock, K. 2001. A study of catches in a fleet of 'ghost-fishing' pots. *Fishery Bulletin* 99: 247–253

Campagna, C., Falabella, V., Lewis, M. 2007. Entanglement of southern elephant seals in squid fishing gear. *Mar. Mamm. Sci.* 23:414–418

CCAMLR. 2012. *Report on Bottom Fisheries and Vulnerable Marine Ecosystems*. Commission on the Conservation of Antarctic Marine Living Resources, North Hobart, Australia.

Chanrachkij, I., Loog-on, A. 2003. *Preliminary Report on Ghost Fishing Phenomena by Drifting FADs in Eastern Indian Ocean*. Southeast Asian Fisheries Development Center, Thailand.

Cheng, I., Chen, T. 1997. Short Note. The incidental capture of five species of sea turtles by coastal setnet [pound net] fisheries in the eastern waters of Taiwan. *Biological Conservation* 82: 235–239.

Chiappone, M., White, A., Swanson, D., Miller, S. 2002. Occurrence and biological impacts of fishing gear and other marine debris in the Florida Keys. *Marine Pollution Bulletin* 44: 597–604

CMS. 2011. *Assessment of Bycatch in Gill Net Fisheries*. By Waugh, S., Filippi, D., Blyth, R., Filippi, P. UNEP/CMS/Inf.10.30. Convention on Migratory Species, Bonn, Germany.

Consoli, P., Sinopoli, M., Deidun, A., Canese, S., Berti, C., Andaloro, F., Romeo, T. 2020. The impact of marine litter from fish aggregation devices on vulnerable marine benthic habitats of the central Mediterranean Sea. *Marine Pollution Bulletin* 152: 110928.

de San, M., Pages, A. 1998. FADs – The western Indian Ocean experience. *SPC Fish Aggregating Device Information Bulletin*: 3: 24-29.

Defaux V., Gascoigne J., Huntington T. 2018. *MSC Pre-assessment of a Ghana Based Pole and Line Tuna Fishery*. Prepared for Thai Union. Poseidon Aquatic Resource Management Ltd., Hampshire, UK.

DelBene, J., Bilkovic, D., Scheld, A. 2019. Examining derelict pot impacts on harvest in a commercial blue crab *Callinectes sapidus* fishery. *Marine Pollution Bulletin* 139:150-156.

Deshponde, P., Philis, G., Brattebo, H., Fet, A. 2020. Using Material Flow Analysis (MFA) to generate the evidence on plastic waste management from commercial fishing gears in Norway. *Resources, Conservation and Recycling*: X 5: doi:10.1016/j.rcrx.2019.100024

Desurmont A, Chapman L. 2000. The use of anchored FADs in the area served by the Secretariat of the Pacific community (SPC): Regional synthesis. IN: Pêche thonière et dispositifs de concentration de poissons, Caribbean-Martinique, 15-19 Oct 1999 15-19 octobre 1999. https://archimer.ifremer.fr/doc/00042/15283/

Donohue, M., Boland, R., Sramek, C., Antonelis, G. 2001. Derelict fishing gear in the Northwestern Hawaiian Islands: diving surveys and debris removal in 1999 confirm threat to coral reef ecosystem. *Mar. Pollut. Bull.* 42: 1301–1312.

DOS. 2020. *Longline Pollution Report*. Digital Observer Services, Erandio, Spain.

Eisenbud, R. 1985. Problems and prospects for the pelagic driftnet. *Boston College Environmental Affairs Law Review* 12: 473-490.

Erzini, K., Monteiro, C., Ribeiro, J., Santos, M., Gaspar, M., Monteiro, P., Borges, T. 1997. An experimental study of gill net and trammel net 'ghost fishing' off the Algarve (southern Portugal). *Marine Ecology Progress Series* 158: 257-265.

Escalle, L., Brouwer, S., Phillips, J., Pilling, G., PNA. 2017. *Preliminary Analyses of PNA FAD Tracking Data from 2016 and 2017*. WCPFC-SC13-2017/MI-WP-05. Kolonia, Federated States of Micronesia, Western and Central Pacific Fisheries Commission

FAO. 2001.*Fishing Gear Types*. Technology Fact Sheets. Food and Agriculture Organization of the United Nations, Rome.

FAO. 2003. *Fishing Techniques*. Technology Fact Sheets. Food and Agriculture Organization of the United Nations, Rome.

FAO. 2019. *FAO Global Fisheries Discards Database*. Available online, (http://www.fao.org/fishery/static/TP633/landdisc.csv. *A Third Assessment of Global Marine Fisheries Discards*. By Roda, M., Gilman, E., Huntington, T., Kennelly, S., Suuronen, P., Chaloupka, M., Medley, P.

FAO Fisheries and Aquaculture Technical Paper 633. ISBN 978-92-5-131226-1. Food and Agriculture Organization of the United Nations, Rome.
Filmalter, J., Capello, M., Deneubourg, J., Cowley, P., Dagorn, L. 2013. Looking behind the curtain: quantifying massive shark mortality in fish aggregating devices. *Frontiers in Ecology and the Environment,* 11: 291-296.
Fitri, A., Pramonowibowo., A. 2015. Economic analysis fyke net modification (case study in Rembang Waters, Central Java). *Indonesian Journal of Fisheries Science and Technology* 11: 1-6.
Fossa, J., Mortensen P., Furevik D. 2002. The deep-water coral *Lophelia pertusa* in Norwegian waters: distribution and fishery impacts. *Hydrobiologia* 471: 1–12.
Fowler, C. 1987. Marine debris and northern fur seals: a case study. *Mar. Pollut. Bull.* 18326–335.
Frisch, A., Baker, R., Hobbs, J., Nankervis, L. 2008. A quantitative comparison of recreational spearfishing and linefishing on the Great Barrier Reef: Implications for management of multi-sector coral reef fisheries. *Coral Reefs* 27: 85-95.
Galbraith, R., Rice, A., Strange, E. 2004. *An Introduction to Commercial Fishing Gear and Methods Used in Scotland*. Fisheries Research Services, Scottish Fisheries Information Pamphlet No. 25. Scottish Executive, Aberdeen.
Gerrodette T, Choy BK, Hlruki L. 1987. *An Experimental Study of Derelict Gill Nets in the Central Pacific Ocean*. SW Fisheries Center, NMFS, Honolulu Laboratory, Admin Rep H-87-18.
GGGI. 2017. *Development of a Best Practice Framework for the Management of Fishing Gear*. By Huntington, T. Global Ghost Gear Initiative, World Animal Protection, London.
Gilardi, K., Carlson-Bremer, D., June, J., Antonelis, K., Broadhurst, G., Cowan, T. 2010. Marine species mortality in derelict fishing nets in Puget Sound, WA and the cost/benefits of derelict net removal. *Marine Pollution Bulletin* 60: 376-382.
Gilman, E. (Ed.). 2009. *Proceedings of the Technical Workshop on Mitigating Sea Turtle Bycatch in Coastal Net and Trap Fisheries*. ISBN: 1-934061-40-9. IUCN, Western Pacific Regional Fishery Management Council, Southeast Asian Fisheries Development Center, Indian Ocean – South-East Asian Marine Turtle MoU, U.S. National Marine Fisheries Service, Southeast Fisheries Science Center, Gland, Switzerland; Honolulu, Bangkok, and Pascagoula, USA.
Gilman, E., Bigler, B., Muller, B., Moreno, G., Largacha, E., Hall, M., Poisson, F., Toole, J., He, P., Chaing, W. 2018. *Stakeholder Views on Methods to Identify Ownership and Track the Position of Drifting Fish Aggregating Devices Used by Tuna Purse Seine Fisheries with Reference to FAO's Draft Guidelines on the Marking of Fishing Gear*. FAO Fisheries Circular 1163. http://www.fao.org/3/BU653en/bu653en.pdf. Food and Agriculture Organization of the United Nations, Rome.
Gilman, E., Castejon, V.D.R., Loganimoce, E., Chaloupka, M. 2020a. Capability of a pilot fisheries electronic monitoring system to meet scientific and compliance monitoring objectives. *Marine Policy* 113: 103792.
Gilman, E., Chaloupka, M., Musyl, M. 2018. Effects of pelagic longline hook size on species- and size-selectivity and survival. *Reviews in Fish Biology and Fisheries* 28: 417-433
Gilman, E., Chaloupka, M., Read, A., Dalzell, P., Holetschek, J., Curtice, C. 2012. Hawaii longline tuna fishery temporal trends in standardized catch rates and length distributions and effects on pelagic and seamount ecosystems. *Aquatic Conservation: Marine and Freshwater Ecosystems* 22: 446-488.
Gilman, E., Chopin, F., Suuronen, P., Kuemlangan, B. 2016. *Abandoned, Lost and Discarded Gillnets and Trammel Nets. Methods to Estimate Ghost Fishing Mortality, and Status of Regional Monitoring and Management*. FAO Fisheries and Aquaculture Technical Paper 600. ISBN 978-92-5-108917-0. Food and Agriculture Organization of the United Nations, Rome.
Gilman, E, Gearhart, J., Price, B., Eckert, S., Milliken, J., Wang, J., Swimmer, Y., Shiode, D., Abe, O., Peckham, S., Chaloupka, M., Hall, M., Mangel, J., Alfaro-Shigueto, J., Dalzell, P., Ishizaki, A. 2010. Mitigating sea turtle bycatch in coastal passive net fisheries. *Fish and Fisheries* 11(1): 57-88.
Gilman, E., Perez-Roda, A., Huntington, T., Kennelly, S., Suuronen, P., Chaloupka, M., Medley, P. 2020. Benchmarking global fisheries discards. *Scientific Reports* 10: 14017.
Gilman, E., Suuronen, P., Hall, M., Kennelly, S. 2013. Causes and methods to estimate cryptic sources of fishing mortality *Journal of Fish Biology* 83: 766-803.
Giordano, S., Lazar, J., Bruce, D., Little, C., Levin, D., Slacum, W.H., Dew-Baxter, J., Methratta, L., Wong, D., Corbin, R., 2010. *Quantifying the Effects of Derelict Fishing Gear in the Maryland Portion of Chesapeake Bay, Final Report to the NOAA Marine Debris Program*. National Oceanic and Atmospheric Administration, Silver Spring, MD
Glass, N., Lavarello, I., Glass, J., Ryan, P. 2000. Longline fishing at Tristan Da Cunha: Impacts on seabirds. Atlantic *Seabirds* 2: 49-56.
Gyi, T. 2020. *Abandoned, Lost or Otherwise Discarded Fishing Gear (ALDFG) in Myanmar's Myeik Archipelago*. Myanmar Ocean Project, Myeik.

Havens, K.J., Bilkovic, D.M., Stanhope, D., Angstadt, K., Hershner, C., 2008. The effects of derelict blue crab traps on marine organisms in the lower York River, Virginia. *North Am. J. Fish. Manage*. 28, 1194–1200
He, P. 2006. Gillnets: Gear design, fishing performance and conservation challenges. *Marine Technology Society Journal* 50: 12-19.
High, W. L., Worlund, D. D. 1979. *Escape of King Crab, Paralithodes camtschatica, from Derelict Pots*. NMFS-SSRF-734.
ICCAT. 2017. *Chair Report of the 1st Joint Tuna RFMO FAD Working Group Meeting*. Joint Tuna RFMO FAD Working Group. International Commission for the Conservation of Atlantic Tunas, Madrid.
ISSF. 2019. *Non-Entangling and Biodegradable FADs Guide*. Version 3. International Seafood Sustainability Foundation, Washington, D.C.
ISSF and IPNLF. 2019. *Skippers' Guidebook to Pole-and-Line Fishing Best Practices*. International Seafood Sustainability Foundation, and International Pole and Line Foundation, Washington, D.C. and London.
Itano, D. 2002. Super Superseiner. In *Proceedings of the 15th Standing Committee on Tuna and Billfish*.
IUCN. 2019. *Expert Survey on Abandoned, Lost and Discarded Gear from Tuna Purse Seine Fisheries*. Survey Records Database. Commission on Ecosystem Management, International Union for the Conservation of Nature, Gland, Switzerland.
James, P., Noble, C., Siikavoupio, S. 2018. *Sea Urchin Fishing Techniques Report*. Nofima, Tromso, Norway.
JBL. 2020. *Spearguns*. Available online, https://www.jblspearguns.com/product/carbine-series/, accessed 5 November 2020. JBL International, Oceanside, California.
Jones, M. 1995. Fishing debris in the Australian marine environment. *Mar. Pollut. Bull*. 30: 25–33.
Karnchanawong, S., Limpiteeprakan, P. 2009. Evaluation of heavy metal leaching from spent household batteries disposed in municipal solid waste. *Waste Management* 29: 550-558.
King Net. 2020. *Purse Seine Net*. Available online, http://www.king-net.com.tw/en/purse-seine-net.html, accessed 13 Sept. 2020. King Net, Kaohsiung, Taiwan.
Law, K. 2017. Plastics in the marine environment. *Annual Review of Marine Science* 9: 205-229.
Lenoir, H., Polet, H., Vanderperren, E. 2019. *Evolution of the Belgian Fisheries*. Institute for Agricultural, Fisheries and Food Research, Oostende, Belgium.
Liu, F., Eugenio, E. 2018. A review and comparison of Bayesian and likelihood-based inferences in beta regression and zero-or-one-inflated beta regression. *Stat Methods Med Research* 27:1024-1044
Lleonart, J., Morales-Nin, B., Massutí, E., Deudero, S., Re~nones, O. 1999. Population dynamics and fishery of dolphinfish (Coryphaena hippurus) in the western Mediterranean. *Sci. Mar*. 63 (3–4), 447–457.
Macfadyen, G., Huntington, T., Cappell, R. 2009. *Abandoned, Lost or Otherwise Discarded Fishing Gear*. UNEP Regional Seas Reports and Studies No.185; FAO Fisheries and Aquaculture Technical Paper No. 523. Rome, UNEP/FAO. 115p.
MacMullen, P., Hareide, N., Furevik, D., Larsson, P., Tschernij, V., Dunlin, G., Revill, A., Pawson, M., Puente, E., Uriarte, A., Sancho, G., Santos, M., Gaspar, M., Erzini, K., Lino, P., Ribeiro, J., Sacchi, J. 2003. *A Study to Identify, Quantify and Ameliorate the Impacts of Static Gear Lost at Sea*. FANTARED 2. ISBN 0-903941-97-X. Sea Fish Industry Authority, Hull.
Macusi, E., Babaran, R., van Zwieten, P. 2015. Strategies and tactics of tuna fishers in the payao (anchored FAD) fishery from general Santos city, Philippines. *Marine Policy* 62: 63-73.
Mahi, I., Baskoro, M., Wisudo, S., Nurani, T., Wiryawan, B. 2018. Supply chain analysis and marketing efficiency of glass eel caught by fyke net (gorong-gorong) in Poso River Estuary, Central Sulawesi, Indonesia. *International Journal of Sciences: Basic and Applied Research* 38: 214-230.
Maryland DNR. 2020. *Pound Net Lighting Requirements*. Available online, https://dnr.maryland.gov/fisheries/pages/poundnets/lighting.aspx, accessed 11 Oct. 2020. Maryland Department of Natural Resources, Annapolis, USA.
Maselko, J., Bishop, G., Murphy, P. 2013. Ghost fishing in the Southeast Alaska commercial Dungeness Crab fishery. *North Am. J. Fish. Manage.* 33, 422–431.
Matthews, T.R., Danson, B., Uhrin, A.V., 2012. *Derelict Trap-Induced Bycatch and Spiny Lobster Mortality in Florida Keys National Marine Sanctuary: Final Report to the NOAA Marine Debris Program*. National Oceanic and Atmospheric Administration, Silver Spring, MD
Maufroy, A., Chassot, E., Joo, R., Kaplan, D. M. 2015. Large-scale examination of spatio-temporal patterns of drifting fish aggregating devices (dFADs) from tropical tuna fisheries of the Indian and Atlantic oceans. *PLoS ONE*, 10, e0128023
Save the Med Foundation. 2020. *Alarming Situation of Ghost Fishing in the Mediterranean*. Save the Med Foundation, Palma, Mallorca, Spain.
Miller, K., Jauharee, A., Nadheeh, I., Adam, M. 2016. *Interactions with Endangered, Threatened, and Protected (ETP) Species in the Maldivian Pole-and-line Tuna Fishery*. International Pole and Line Foundation, London, and Marine Research Centre, Male, Maldives.

Miller, K., Nadheeh, I., Jauharee, A., Anderson, R., Adam, M. 2017. Bycatch in the Maldivian pole-and-line tuna fishery. *PLoS ONE* 12: e0177391.
Mio, S., Domon, T., Yoshida, K., Matsumura, S. 1990. Preliminary study on change in shape of drifting nets placed in the sea. In: Shornura RS. Godfrey ML (eds) *Proceedings of the Second International Conference on Marine Debris*. 2-7 April 1989, Honolulu, Hawaii. US Dept Commerce, NOAA Tech Memo NMFS, NOAA-TM-NMFS-SWFSC-154.
Munoz, P., Murillo, F., Sayago-Gil, M., et al. 2011. Effects of deep-sea bottom longlining on the Hatton Bank fish communities and benthic ecosystem, north-east Atlantic. *Journal of the Marine Biological Association of the United Kingdom* 91: 939-952.
Mustad Autoline. 2020. *Mustad Autoline Snooded Hooks*. Available online, https://mustadautoline.com/products/mustad-autoline-consumables/snooded-hooks, 21 Sept. 2020. Mustad, Gjøvik, Norway.
Natural Resources Consultants. 1990. *Survey and Evaluation of Fishing Gear Loss in Marine and Great Lakes Fisheries of the United States*. Final Report Prepared for the National Marine Fisheries Service Under Contract 50ABNF-9-00144. Natural Resources Consultants, Inc., Seattle, Washington.
Netmark. 2020. *Nettings. Purse Seining. Nettings of Twisted and Braided Nylon*. Available online, https://netmarkas.com/purse-seining/, accessed 13 Sept. 2020. Netmark, Fredericia, Denmark.
Ningtiyas, S., Rachman, F., Setyobudi, E., Djumanto, R., Djasmani, S. 2020. The effect of bait types on the catch of foldable dome fishing pots operated in Tuban regency. *IOP Conference Series Earth and Environmental Sciences* 404: doi: 10.1088/1755-1315/404/1/012077.
ODFW. 2019. *Commercial Sea Urchin Fishing*. Available online, https://www.dfw.state.or.us/MRP/shellfish/commercial/urchin/index.asp, accessed 5 Nov. 2020. Oregon Department of Fish and Wildlife, Salem, Oregon.
Oksanen SM, Ahola MP, Oikarinen J, Kunnasranta M. 2015. A novel tool to mitigate by-catch mortality of Baltic seals in coastal fyke net fishery. *PLoS ONE* 10(5): e0127510.
Okuda, T., Nishida, T. 2017. *Provisional Bottom Fishing Impact Assessment for Japanese Midwater Trawl Fisheries in SIOFA Convention Area*. SC-03-06.2(02). Southern Indian Ocean Fisheries Agreement, Saint-Denis, Reunion.
O'Neill, F., Noack, T. 2020. The geometry and dynamics of Danish anchor seine ropes on the seabed. ICES *Journal of Marine Science*: doi:10.1093/icesjms/fsaa198.
Pacific Ocean Producers. 2019. *POP Fishing and Marine Commercial Fishing Catalog*. Pacific Ocean Producers, Honolulu.
Perez-Roda, M., Gilman, E., Huntington, T., Kennelly, S., Suuronen, P., Chaloupka, M., Medley, P. 2019. *A Third Assessment of Global Marine Fisheries Discards*. FAO Fisheries and Aquaculture Technical Paper 633. ISBN 978-92-5-131226-1. Food and Aquaculture Organization of the United Nations, Rome.
Prado, J. 1990. *Fisherman's Workbook*. ISBN 0-85238-163-8. Food and Agriculture Organization of the United Nations, Rome, and Fishing New Books, Oxford.
Prajith, K., Remesan, M. 2019. Design, construction and operation of fishing pots and traps. IN: Edwin, L., Thomas, S., Remesan, M., Ashraf, P., Baiju, M., Lekshmi, N., Madhu, V.R. (Eds.). *ICAR Winter School Manual- Responsible Fishing: Recent Advances in Resource and Energy Conservation*. Central Institute of Fisheries Technology, Kochi, India, pp. 319-326.
Proctor, C., Natsir, M., Mahiswara, et al. 2019. *A Characterisation of FAD-Based Tuna Fisheries in Indonesian Waters*. ACIAR Project FIS/2009/059. ISBN: 978-0-646-80326-5. Australian Centre for International Agricultural Research, Canberra.
Renchen, G., Pittman, S.J., Clark, R., Caldow, C., Gall, S., Olsen, D., Hill, R. 2014. Impact of derelict fish traps in Caribbean waters: an experimental approach. *Bull. Mar. Sci*. 90, 551–563
Revill, A., Dunlin, G. 2003. The fishing capacity of gillnets lost on wrecks and on open ground in UK coastal waters. *Fish Res.* 64:107-113
Rouxel, Y. 2017. *Best Practices for Fishing Sustainability: Fishing Gear Assessment in the Newfoundland Inshore Northern Cod Fishery*. MSc Thesis. University of Akureyri, Isafjordur, Iceland.
Sala, A., Brcic, J., Conides, A., et al. 2013. *Final Project Report. Technical Specifications of Mediterranean Trawl Gears*. Evento, organizzato dall'Istituto di Scienze Marine del Consiglio Nazionale delle Ricerche (CNR-ISMAR), Ancona, Italy.
Samoilys, M., Maina, G., Osuka, K. 2011. *Artisanal Fishing Gears of the Kenyan Coast*. ISBN: 978-9966-21-115-6. CORDIO and USAID, Mombasa.
Scheld, A., Bilkovic, D., Havens, K. 2016. The dilemma of derelict gear. *Scientific Reports*, *6*, 19671.
Seattle Marine and Fishing Supply. 2020. *Commercial Fishing Gear Catalogue*. Seattle Marine and Fishing Supply, Seattle.

Shainee, M., Leira, B. 2011. On the cause of premature FAD loss in the Maldives. *Fisheries Research* 109: 42–53.
Sibisopere M. 2000. The significant contribution of FADs to Solomon Taiyo Limited's fishing operations. *Pêche thonière et dispositifs de concentration de poissons, Caribbean-Martinique, 15-19 Oct 1999*. https://archimer.ifremer.fr/doc/00042/15307/
Silva, D. R., J. T. DeAlteris, H. O. Milliken. 2011. Evaluation of a pound net leader designed to reduce sea turtle bycatch. *Marine Fisheries Review* 73: 36-45.
Sinopoli, M., Cillari, T., Andaloro, F., Berti, C., Consoli, P., Galgani, F., Romeo, T. 2020. Are FADs a significant source of marine litter? Assessment of released debris and mitigation strategy in the Mediterranean Sea. *Journal of Environmental Management* 253: 109749.
Slack-Smith, R. 2001. *Fishing with Traps and Pots*. FAO Training Series 26. Available online, http://www.fao.org/3/x2590e/x2590e00.htm, accessed 10 Oct. 2020. Food and Agriculture Organization of the United Nations, Rome.
Stelfox, M., Hudgins, J., Ali, K., Anderson, R. 2014. *High mortality of Olive Ridley Turtles (Lepidochelys olivacea) in ghost nets in the central Indian Ocean.* WPEB10-28. Indian Ocean Tuna Commission, Mahe, Seychelles.
Stelfox, M., Lett, C., Reid, G., Souch, G., Sweet, M. 2020. Minimum drift times infer trajectories of ghost nets found in the Maldives. *Marine Pollution Bulletin* 154: 111037.
Stevens, B. 2020. The ups and downs of traps: environmental impacts, entanglement, mitigation, and the future of trap fishing for crustaceans and fish. *ICES Journal of Marine Science* doi: 10.1093/icesjms/fsaa135.
Stevens, B.G., Vining, I., Byersdorfer, S., Donaldson, W., 2000. Ghost fishing by Tanner crab (*Chionoecetes bairdi*) pots off Kodiak, Alaska: pot density and catch per trap as determined from sidescan sonar and pot recovery data. *Fish. Bull*. 98, 389–399.
Stevens, B.G. 2014. Impacts of fishing on king crabs: bycatch, injuries, and mortality. In *King Crabs of the World: Biology and Fisheries Management*, pp. 363–402. Ed. by B. G. Stevens. CRC Press (Taylor and Francis), Boca Raton, FL
Sukhsangchan, C., Phuynoi, S., Monthum, Y., Whanpetch, N., Kulanujaree, N. 2020. Catch composition and estimated economic impacts of ghost-fishing squid traps near Suan Son Beach, Rayong province, Thailand. *Science Asia* 46: 87-92.
Suuronen, P., Chopin, F., Glass, C., Lokkeborg, S., Matsushita, Y., Queirolo, D., Rihan, D. 2012. Low impact and fuel-efficient fishing – looking beyond the horizon. *Fish. Res*. 119–120: 135–146.
Suuronen, P., Pitcher, C., McConnaughey, R., Kaiser, M., Hiddink, J., Hilborn, R. 2020. A path to a sustainable trawl fishery in southeast Asia. *Reviews in Fisheries Science & Aquaculture* 28: 499-517.
Tang H, Xu L, Hu F. 2018. Hydrodynamic characteristics of knotted and knotless purse seine netting panels as determined in a flume tank. *PLoS ONE* 13: e0192206.
Tasliel, A. 2008. *Determination of Amount of Lost Fishing Gear on Karatas and Yumurtalik (Iskenderun Bay) During a Fishing Season*. MSc Thesis. Department of Fisheries, Institute of Naturel and Applied Sciences, University of Cukurova, Adana, Turkey.
Thai Union. 2017. *Marine Stewardship Council Pre-Assessment of the Pole and Line Fishery in Senegal*. Thai Union Europe, Paris.
Tietze, U., Lee, R., Siar, S., Moth-Poulsen, T., Bage, H. 2011. *Fishing with Beach Seines*. FAO Fisheries and Aquaculture Technical Paper 562. Food and Agriculture Organization of the United Nations, Rome.
Touhy, A., Skalski, J., Jorgenson, A. 2020. Modified commercial fish trap to help eliminate salmonid bycatch mortality. *North American Journal of Fisheries Management* DOI: 10.1002/nafm.10496
Tuna Fish Tackle. 2020. *Harpoons*. Available online, https://www.tunafishtackle.com/product-category/gaffs-harpoons/harpoons/, accessed 5 November 2020. Tuna Fish Tackle, West Babylon, New York.
Uhrin, A., Matthews, T., Lewis, C. 2014. Lobster trap debris in the Florida Keys National Marine Sanctuary: distribution, abundance, density, and patterns of accumulation. *Marine Coastal Fisheries*, 20–32
Uhrin AV, Schellinger J. 2011. Marine debris impacts to a tidal fringing-marsh in North Carolina. *Mar. Pollut. Bull.* 62:2 605–2610.
UNEP MAP. 2015. *Regional Survey on Abandoned, Lost or Discarded Fishing Gear & Ghost Nets in the Mediterranean Sea*. Available online, https://mio-ecsde.org/wp-content/uploads/2016/12/ALDFG-in-the-MED.pdf. United Nations Environment Programme, Mediterranean Action Plan, Athens, Greece.
Uzer, U., Yildiz, T., Karakulak, F. 2017. Catch composition and discards of the boat seine in the Istanbul Strait (Turkey). *Turkish Journal of Zoology* 41: 702-713.
Voss, C.M., Wood, A., Browder, J.A., Michaelis, A. 2012. *Estimating Derelict Crab Pot Density and Bycatch in North Carolina, Final Report to the NOAA Marine Debris Program*. National Oceanic and Atmospheric Administration, Silver Spring, MD

Watson, R. 2017. A database of global marine commercial, small-scale, illegal and unreported fisheries catch 1950-2014. Available online, http://dx.doi.org/10.4226/77/5a65572655f73, accessed 20 June 2020. *Scientific Data* 4: 170039. DOI: 10.1038/sdata.2017.39.

WCPFC. 2019. *Conservation and Management Measure for Bigeye, Yellowfin and Skipjack Tuna in the Western and Central Pacific Ocean*. CMM 2018-01. Western and Central Pacific Fisheries Commission, Kolonia, Federated States of Micronesia.

Webber D, Parker S. 2012. Estimating unaccounted fishing mortality in the Ross Sea region and Amundsen Sea (CCAMLR subareas 88.1 and 88.2) bottom longline fisheries targeting Antarctic toothfish. *CCAMLR Science* 19: 17-30.

Widodo, A., Wudianto, X., Satria, F. 2016. Current status of the pole-and-line fishery in eastern part of Indonesia. *Indonesian Fisheries Research Journal* 22: 43-52.

Wilcox, C., Heathcote, G., Goldberg, J., Gunn, R., Peel, D., Hardesty, B. 2014. Understanding the sources and effects of abandoned, lost, and discarded fishing gear on marine turtles in northern Australia. *Conservation Biology* 29: 198-206.

Yildiz, T., Karakulak, F. 2016. Types and extent of fishing gear losses and their causes in the artisanal fisheries of Istanbul, Turkey. *Journal of Applied Ichthyology* 32, 3.

Zabka, T., Haulena, M., Puschner, B., Gulland, F., Conrad, P., Lowenstine, L. 2006. Acute lead toxicosis in a harbor seal (Phoca vitulina richardsi) consequent to ingestion of a lead fishing sinker. *Journal of Wildlife Diseases* 42: 651-657.

Zhou, C., Zu, L., Tang, H., Hu, F., He, P., Kumazawa, T., Wang, X., Wan, R., Dong, S. 2019. Identifying the design alternatives and flow interference of tuna purse seine by the numerical modelling approach. *Journal of Marine Science and Engineering* 7: doi:10.3390/jmse7110405.
